# Dynamics of skeletal muscle-resident stem cells during myogenesis in fibrodysplasia ossificans progressiva

**DOI:** 10.1038/s41536-021-00201-8

**Published:** 2022-01-14

**Authors:** Alexandra Stanley, Elisia D. Tichy, Jacob Kocan, Douglas W. Roberts, Eileen M. Shore, Foteini Mourkioti

**Affiliations:** 1 Department of Orthopaedic Surgery, Perelman School of Medicine, University of Pennsylvania, Philadelphia, PA 19104 USA; 2 Center for Research in FOP and Related Disorders, Perelman School of Medicine, University of Pennsylvania, Philadelphia, PA 19104 USA; 3 Department of Genetics, Perelman School of Medicine, University of Pennsylvania, Philadelphia, PA 19104 USA; 4 Penn Institute for Regenerative Medicine, Musculoskeletal Program, Perelman School of Medicine, University of Pennsylvania, Philadelphia, PA 19104 USA; 5 Department of Cell and Developmental Biology, Perelman School of Medicine, University of Pennsylvania, Philadelphia, PA 19104 USA

**Keywords:** Muscle stem cells, Diseases

## Abstract

Fibrodysplasia ossificans progressiva (FOP) is a rare genetic disease in which extraskeletal (heterotopic) bone forms within tissues such as skeletal muscles, often in response to injury. Mutations in the BMP type I receptor ACVR1/ALK2 cause FOP by increasing BMP pathway signaling. In contrast to the growing understanding of the inappropriate formation of bone tissue within the muscle in FOP, much is still unknown about the regenerative capacity of adult diseased muscles. Utilizing an inducible *ACVR1*^*R206H*^ knock-in mouse, we found that injured *Acvr1*^*R206H/+*^ skeletal muscle tissue regenerates poorly. We demonstrated that while two resident stem cell populations, muscle stem cells (MuSCs) and fibro/adipogenic progenitors (FAPs), have similar proliferation rates after injury, the differentiation potential of mutant MuSCs is compromised. Although MuSC-specific deletion of the *ACVR1*^*R206H*^ mutation does not alter the regenerative potential of skeletal muscles in vivo, *Acvr1*^*R206H/+*^ MuSCs form underdeveloped fibers that fail to fuse in vitro. We further determined that FAPs from *Acvr1*^*R206H/+*^ mice repress the MuSC-mediated formation of *Acvr1*^*R206H/+*^ myotubes in vitro. These results identify a previously unrecognized role for *ACVR1*^*R206H*^ in myogenesis in FOP, via improper interaction of tissue-resident stem cells during skeletal muscle regeneration.

## Introduction

Fibrodysplasia ossificans progressiva (FOP) is a rare genetic disease in which heterotopic (extraskeletal) bone forms within skeletal muscle and other soft connective tissues^1,2^. This bone is qualitatively normal, but forms in abnormal locations in the body. Mutations in the type l bone morphogenetic protein (BMP) activin A receptor type 1 (ACVR1) are the cause of FOP, with the R206H (c.617 G > A) mutation as the most prevalent^2,3^. This mutation increases both BMP ligand-dependent and ligand-independent signaling to promote downstream chondro/osteogenic gene expression and heterotopic ossification (HO) formation in FOP patients^4^. HO formation can occur spontaneously, but it is frequently induced by injury to skeletal muscle^1^. HO can also occur in the absence of a known genetic mutation in response to severe tissue trauma, such as from high impact blast injury, spinal cord injury, or joint-replacement surgery^5^; therefore, understanding the associated effects on tissue regenerative mechanisms is highly relevant for both FOP patients, as well as the general population.

While the origin of the heterotopic bone tissue has been extensively studied^6–8^, the impact of the *ACVR1*^*R206H*^ mutation on muscle regeneration capacity remains unclear. It is possible that the diversion of skeletal muscle and other soft connective tissues towards bone formation could be due in part to improper tissue repair following injury. Muscle injury, as a common trigger of heterotopic bone in FOP patients^1^, is indicative of an aberrant skeletal muscle regeneration response to the *ACVR1* mutation. We have previously reported that expression of *Acvr1*^*R206H/+*^ in knock-in mouse models of FOP recapitulates all key clinical features of the disease and induces HO in response to muscle injury^9^, supporting that an inducible knock-in mouse could serve as an appropriate model to investigate muscle regeneration in adult mice.

Skeletal muscle is a highly regenerative tissue after damage. Muscle stem cells (MuSCs; also known as satellite cells) are required for the repair of damaged myofibers^10^. These cells lie quiescent within the basal lamina of the fiber until stimulated to activate following tissue injury. MuSCs then begin the process of proliferation and self-renewal, increasing their numbers to efficiently differentiate and fuse to form myofibers^10^. Delays in and/or improper gene expression of the myogenic program after injury are detrimental for muscle regeneration^11^. Fibro/adipogenic progenitor cells (FAPs) are another skeletal muscle-resident progenitor cell type that, while not inherently myogenic, are necessary for myogenic progression^12–19^. Evidence supports that FAPs have a role in supporting the regenerative potential of dystrophic and aged muscles^12–14,19,20^. Moreover, in some disease settings, FAPs have been implicated as the source for ectopic fibrotic tissue, fat, cartilage, and bone^6,12,17,21^. It was previously shown that the FOP mutation does not alter muscle morphology during development^6^; however, there is evidence of muscle atrophy and weakness in mature skeletal muscle tissue in FOP patients^3,22^. The effect of the *ACVR1*^*R206H*^ mutation in adult MuSCs and FAPs post-injury has not been investigated in the context of FOP. Accordingly, in this study, we sought to clarify the impact of the *ACVR1*^*R206H*^ mutation on muscle regeneration, particularly in regard to the functionality of muscle-resident progenitor cells during myogenesis. We report that under the influence of the *ACVR1*^*R206H*^ FOP mutation, MuSC formation of myofibers is impaired and FAPs are unable to support MuSC myogenic potential. These data reveal a fundamental role of FAPs in influencing the myogenic activity of *Acvr1*^*R206H/+*^ MuSCs after injury, and, as a consequence, MuSC-FAP coordination may be an important target for future therapeutic interventions to improve the health of those with FOP.

## Results

### Acvr1^R206H/+^ skeletal muscle tissue does not repair properly after injury

**Fig. 1:**
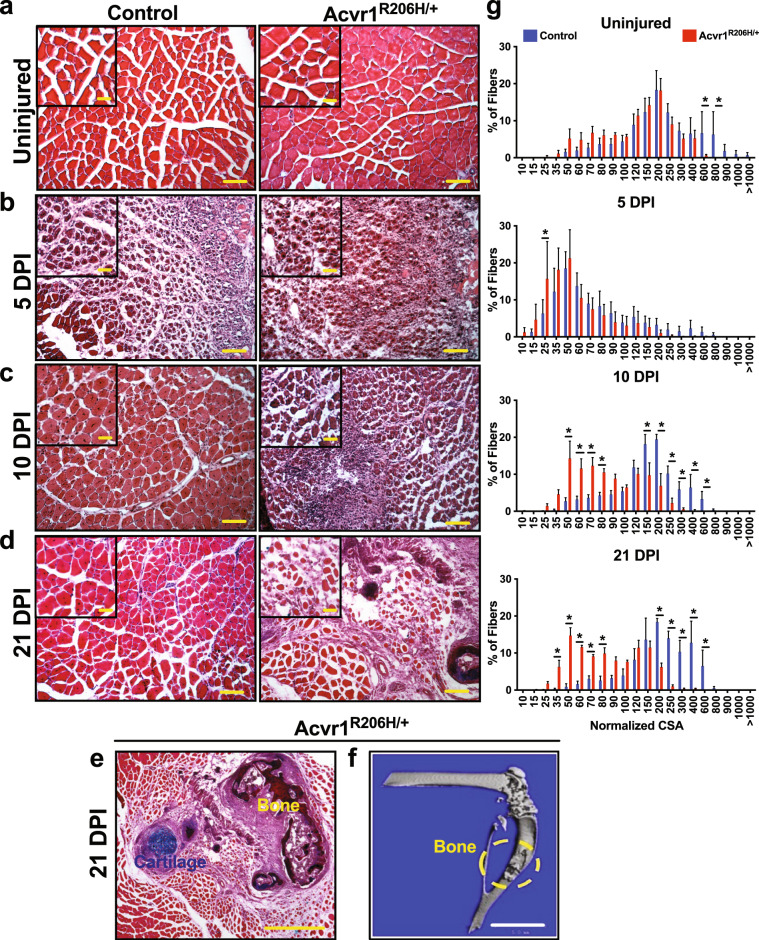
*Acvr1*^*R206H/+*^ skeletal muscle tissue does not repair properly after injury. **a** Prior to injury, fibers from skeletal muscle tissue from *Acvr1*^*R206H/+*^ mice are slightly smaller compared to control tissue. **b–e** Control and *Acvr1*^*R206H/+*^ mice were subjected to Tibialis Anterior (TA) injury with CTX. Representative images of H&E staining of skeletal muscle sections at timepoints after injury. **b** At 5 DPI, control muscle shows fibers with centralized nuclei, a sign of repairing fibers. *Acvr1*^*R206H/+*^ muscle appears fibrotic with fewer repairing fibers. **c** At 10 DPI, control muscle has continued to repair, while *Acvr1*^*R206H/+*^ muscle tissue appears more fibrotic and muscle damage persists. **d, e** At 21 DPI, control muscle had fully repaired, while ectopic bone with adjacent regions of mature cartilage (as shown by Alcian blue hematoxylin/orange G stain) was present in *Acvr1*^*R206H/+*^ muscle. Scale bar = 200 µm for **a–c** images; 500 µm for **d** images; 100 µm for **e** image; 20 µm for all inset images**. f** Representative µCT image of HO in Tibialis Anterior (TA) muscle (circled in yellow). Scale bar = 5 mm. **g** Quantification of fiber size (normalized cross-sectional area; CSA) of regenerating fibers (µm^2^) during regeneration (timepoints are indicated on graphs). *n* = 3–5 mice for each timepoint. Statistical significance was determined by student’s *t*-test; *p < 0.05.

Intramuscular injection of cardiotoxin (CTX) is widely used to study the events and molecular players involved in acute skeletal muscle regeneration^23^. To examine the effects of the *ACVR1*^*R206H*^ mutation on skeletal muscle regeneration kinetics, CTX-injured tibialis anterior (TA) muscles from *Acvr1*^*R206H/+*^ and control (*Acvr1*^*+/+*^) mice were harvested at timepoints post-injury. Prior to injury, *Acvr1*^*R206H/+*^ fibers trend slightly smaller on average but were similar in morphology compared to controls (Fig. 1a, g). However, after injury *Acvr1*^*R206H/+*^ muscle tissue had smaller regenerating myofibers relative to controls, as marked by fibers with centralized nuclei at both day 5- and 10-days post-injury (DPI) (Fig. 1b, c, and g). By 21-days post-injury, cartilage along with the persistence of damaged muscle was present in *Acvr1*^*R206H/+*^ tissue, compared to the proper repair observed in control muscle tissue (Fig. 1d, e, and g). In response to CTX injury, HO was evident by 21 days (Fig. 1e and f), confirming that heterotopic endochondral bone forms in response to injury in *Acvr1*^*R206H/+*^ mice. Interestingly, mutant muscles at 21 DPI exhibit a smaller fiber area compared to controls (Fig. 1g), suggesting a continued delay in myofiber regeneration. These data indicate that along with the ectopic bone lesions, there is a previously unrecognized delay and inefficient repair of skeletal muscle tissue after injury.

### Comparable in vivo proliferation capability between control and Acvr1^R206H/+^ MuSCs and FAPs

MuSCs are required for skeletal muscle regeneration^10^. MuSCs become activated shortly after skeletal muscle injury induced by CTX, with cell numbers peaking at 3–4 days after injury, and then decreasing during differentiation and maturation of cells until muscle architecture is restored (14–21 days after injury)^24,25^. The *ACVR1*^*R206H*^ mutation increases BMP pathway signaling, and BMP signaling has been suggested to influence cell proliferation^26^. On the other hand, fibro/adipogenic cells (FAPs), a muscle tissue-resident mesenchymal stem cell population, increase ~2–3 days after injury, with their numbers returning to pre-injury levels about 4–5 days after injury^24^. To evaluate the level of BMP signaling in diseased cells, MuSCs and FAPs were isolated by fluorescent activated cell sorting (FACS) (Supplementary Figs. 1 and 2), immunostained for the BMP receptor-mediated phosphorylation of transcription factors Smad1, 5, and 8 (collectively pSmad1/5/8). We found elevated levels in both MuSCs and FAPs isolated from *Acvr1*^*R206H/+*^ compared to control mice (Supplementary Fig. 3). These data confirmed that the ACVR1 mutation induces increased BMP activity through the pSmad1/5/8 signaling in MuSCs and FAPs.

**Fig. 2:**
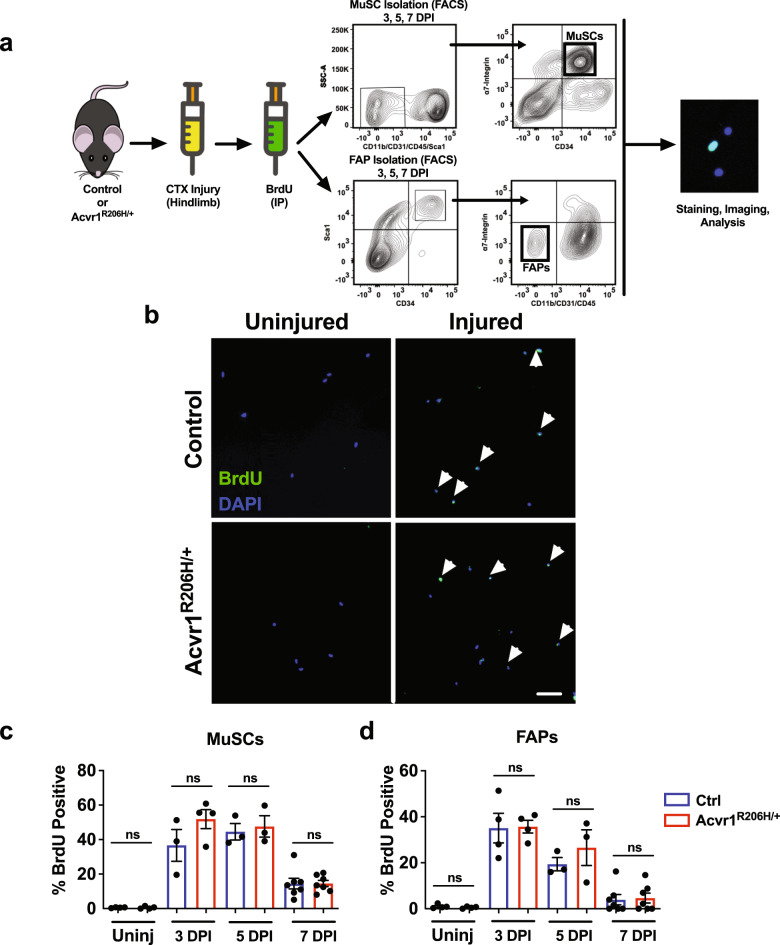
In vivo proliferation capability is similar between control and *Acvr1*^*R206H/+*^ MuSCs and FAPs. **a** Schematic representation of in vivo proliferation assay. The Tibialis Anterior and Gastrocnemius muscles of mice (2 month old) were injured with CTX and intraperitoneally injected with BrdU to label proliferating cells 24 h before isolation. MuSCs and FAPs were isolated by fluorescent activated cell sorting (FACS) based on the cell surface markers CD34 and α7-integrin for MuSCs and Sca1, CD34, and lack of α7-integrin for FAPs. **b** Representative images of BrdU-stained (green) isolated control and *Acvr1*^*R206H/+*^ cells from uninjured and injured muscle. Scale bar = 50 μm. **c** Quantification of the percent of BrdU^+^ MuSCs and **d** FAPs. Graphs represent the mean ± SEM. *n* ≥ 3 mice for each group; *N* > 300 MuSCs or FAPs per timepoint and genotype were analyzed. Statistical significance was determined by one-way ANOVA; ns not significant.

To address whether the poor muscle regeneration we observed in *Acvr1*^*R206H/+*^ mutants could be a result of proliferative defects, we assessed MuSC and FAP proliferation in vivo. Control and *Acvr1*^*R206H/+*^ mice were injured with CTX, and then pulsed at 2, 4, or 6 days after injury with an intra-peritoneal injection of 5-bromo-2’-deoxy-uridine (BrdU) (Fig. 2a). MuSCs and FAPs were FACS-isolated 24 h after BrdU injection (at 3, 5, and 7 days after CTX injury) and examined for the proportion of nuclei that incorporated BrdU (Fig. 2b). In both uninjured control and *Acvr1*^*R206H/+*^ mice, only a small portion of MuSCs were BrdU^+^ (Fig. 2c), consistent with adult MuSCs quiescence under steady-state conditions^10^. At days 3 and 5 post-CTX injury, both genotypes showed a similar increase in the percentage of proliferating MuSCs (Fig. 2c). At day 7 post-CTX injury, similarly reduced numbers of proliferating MuSCs were apparent in both control and *Acvr1*^*R206H/+*^ skeletal muscle tissues (Fig. 2c), consistent with previous reports that demonstrated that a majority of MuSCs are differentiating at this time to facilitate muscle regeneration, as well as having replenished the stem cell pool^24^. Overall, the comparable rates of MuSC proliferation in control and *Acvr1*^*R206H/+*^ mice in vivo (Fig. 2c) suggest that MuSCs are activated and enter the cell cycle at similar levels after injury. Similarly, we did not detect significant differences in the proliferation rates of FAPs between controls and *Acvr1*^*R206H/+*^ cells (Fig. 2d), indicating that the observed muscle phenotype is not due to a proliferation defect of this lineage. These data demonstrate that MuSCs and FAPs are both present and capable of proliferating efficiently in response to injury in *Acvr1*^*R206H/+*^ skeletal muscles.

### Acvr1^R206H/+^ FAPs fail to decline during regeneration

**Fig. 3:**
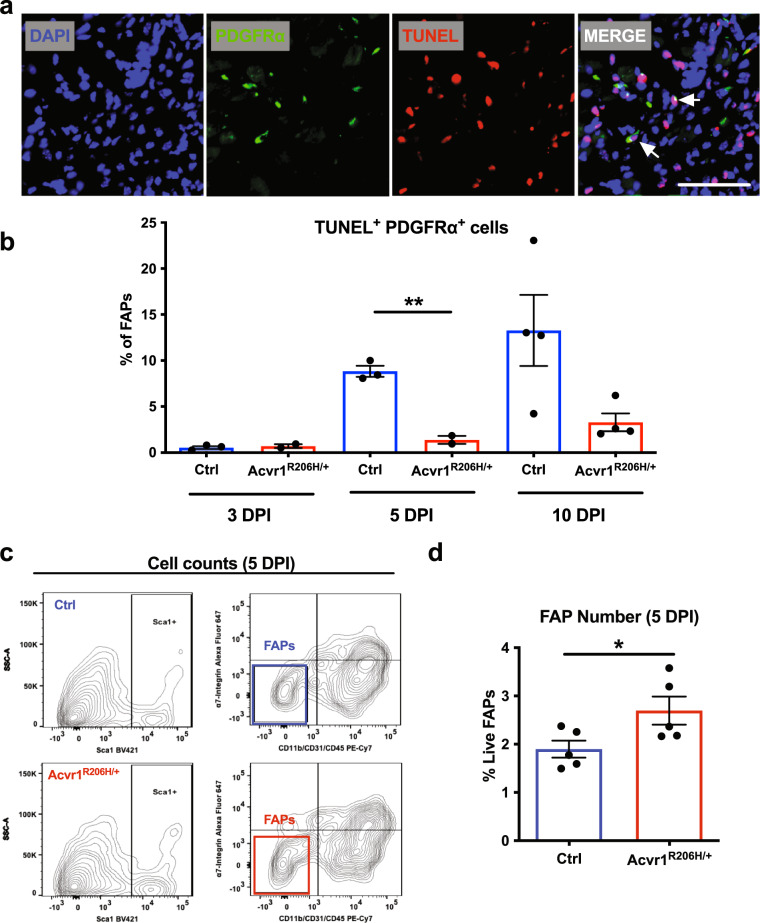
*Acvr1*^*R206H/+*^ FAPs fail to decline during regeneration. **a** Representative images of control and *Acvr1*^*R206H/+*^ cryosections stained with the FAP marker PDGFRα (green) and DAPI (nuclei; blue), and processed for cell death by TUNEL (red). Scale bar = 50 μm. **b** Analysis of TUNEL positive FAPs at 3-, 5-, and 10- days post-CTX injury. *N* = 2–5 mice per condition per genotype. Mean ± SEM displayed. Statistical significance was determined by unpaired student *t*-tests with Welch’s correction. **p ≤ 0.01. **c** Flow cytometry scatter plots of FAPs from 5 DPI mice. **d** Analysis of FAP numbers by flow cytometry. Mean ± SEM displayed. Significance was determined by student *t*-tests. *p ≤ 0.05. *n* = 5–6 mice per genotype.

Myogenesis depends on the proper and coordinated communication between endogenous progenitor cell populations^19^. Loss of MuSCs has been shown to result in altered myogenic programming and failed regeneration^11^, while an excess of FAPs at the later regeneration stages has been proposed to contribute to tissue degeneration during chronic muscle injuries^27^. Therefore, we sought to determine whether delayed muscle regeneration in injured *Acvr1*^*R206H/+*^ skeletal muscle tissue is associated with either loss of MuSCs post-injury and/or defects of the physiological apoptosis rates of FAPs. Control and *Acvr1*^*R206H/+*^ mice were CTX-injured, and muscles were examined at days 3, 5, and 10 post-injury. Cell death was assessed by TUNEL staining of cryosections. MuSCs were identified by the muscle stem cell marker Pax7^28^, while FAPs were identified with the platelet derived growth factor receptor alpha (PDGFRα) marker, as previously shown to label these cells^13^. This analysis revealed no significant increase in cell death of *Acvr1*^*R206H/+*^ MuSCs at any timepoint, relative to controls (Supplementary Fig. 4). However, we found significantly decreased levels of apoptosis (TUNEL^+^ Pdgfrα^+^) of FAPs at 5 DPI (Fig. 3a, b), suggesting that a higher number of FAPs persist in the FOP damaged muscles compared to controls. To further examine whether FAP numbers persist following injury, we compared FAP numbers by flow cytometry 5 days post-injury and found continued FAP accumulation in *Acvr1*^*R206H/+*^ muscles compared to controls (Fig. 3c, d), consistent with an impaired FAP clearance during skeletal muscle regeneration in FOP.

Altogether, these data demonstrate that the muscle regenerative failure seen in *Acvr1*^*R206H/+*^ mice is not the result of MuSC loss due to cell death, but instead, may be attributed to aberrantly persistent numbers of FAPs that remain in *Acvr1*^*R206H/+*^ injured muscles during late phases of regeneration. These results are consistent with the idea that excess FAPs in FOP not only contribute to the ectopic bone as progenitor cells^6^ but also influence myogenesis of *Acvr1*^*R206H/+*^ MuSCs.

### Acvr1^R206H/+^ activated MuSCs fail to form mature myofibers

**Fig. 4:**
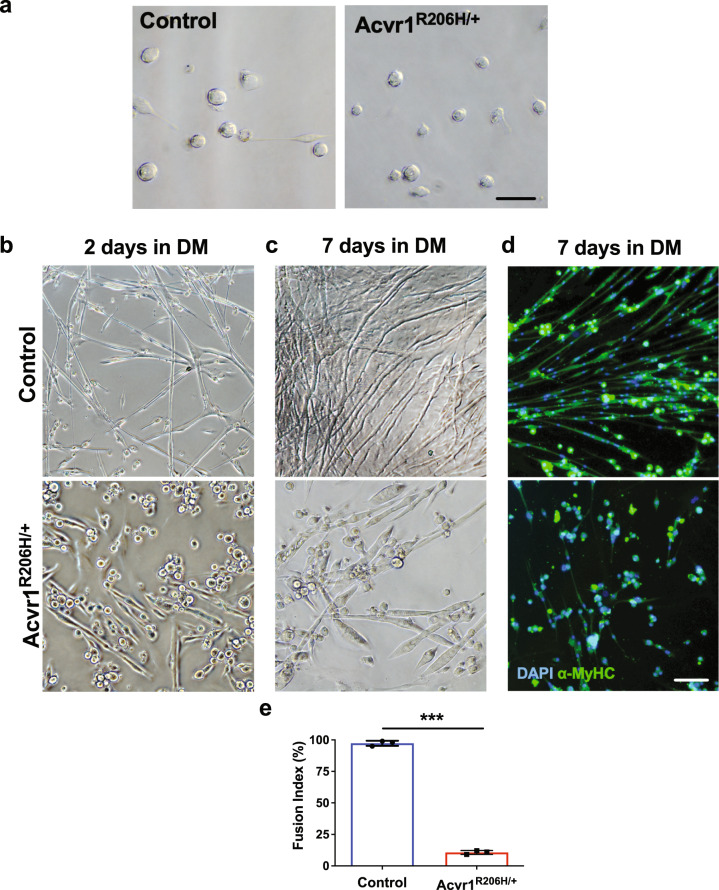
*Acvr1*^*R206H/+*^ MuSCs fail to form properly fused myofibers. **a** Representative images show that freshly isolated MuSCs from control and *Acvr1*^*R206H/+*^ skeletal muscle have similar morphologies. **b–d** Representative images of control MuSCs after 2 days (**b** top) and 7 days (**c** top) in differentiation media (DM) show fusion into myotubes and normal myofibers are formed. Representative images of *Acvr1*^*R206H/+*^ MuSCs after 2 days (**b** bottom) and 7 days (**c** bottom) in DM show that *Acvr1*^*R206H/+*^ cells do not differentiate into normal myofibers and have reduced fusogenic efficiency. **d** Representative images of control and *Acvr1*^*R206H/+*^ MuSCs that were stained for the mature muscle marker alpha-myosin heavy chain (α-MyHC) after 7 days in myogenic differentiation media are shown. **e** Quantification of fusion index (percent of total nuclei residing in cells with 3 or more nuclei) after 7 days in culture. All data are expressed as mean ± SEM*;* Scale bars for all images = 100 µm. *n* ≥ 3 mice for each group; *N* > 100 MuSCs (nuclei) per timepoint and genotype were analyzed. Statistical significance was determined by one-way ANOVA, ***p < 0.0001.

At later stages of regeneration, myogenic precursors withdraw from the cell cycle and differentiate into mature myofibers^10^. Since MuSCs from *Acvr1*^*R206H/+*^ skeletal muscle tissue proliferate normally the following injury, we next investigated their ability to differentiate utilizing in vitro assays. Similar abundances of MuSCs from uninjured control and *Acvr1*^*R206H/+*^ mice were identified and isolated by FACS (Supplementary Fig. 1). We found that both control and *Acvr1*^*R206H/+*^ MuSCs had similar morphology immediately following isolation (Fig. 4a). However, after 2 days in standard myogenic differentiation medium (DM), control MuSCs fused to form elongated and branching myofibers (Fig. 4b, top), while *Acvr1*^*R206H/+*^ MuSCs did not fuse as readily and did not form extended or branching myofibers (Fig. 4b, bottom). Furthermore, while control myofibers continued to branch and mature over time (Fig. 4c, top), after 7 days *Acvr1*^*R206H/+*^ myofibers remained shorter and wider, with no branches formed (Fig. 4c, bottom). To further examine the maturity of myofibers in vitro, day 7 cultures were stained for the late myogenic differentiation marker α-myosin heavy chain (α-MyHC)^29^. Reduced α-MyHC expression was detected in *Acvr1*^*R206H/+*^ cells as compared to controls (Fig. 4d). As an additional measure of myogenic differentiation, the fusion index was calculated as the percentage of total nuclei that resided in cells containing 3 or more nuclei. The fusion index for *Acvr1*^*R206H/+*^ MuSCs after 7 days in myogenic media was significantly lower than controls (Fig. 4e), further indicating a delayed or impaired differentiation capacity of *Acvr1*^*R206H/+*^ MuSCs to form mature multinucleated myofibers in vitro. These data demonstrate that increased BMP signaling, as a result of increased ACVR1 activity, leads to deficient differentiation capacity of *Acvr1*^*R206H/+*^ MuSCs in vitro.

### Impaired differentiation of Acvr1^R206H/+^ MuSCs in vivo

**Fig. 5:**
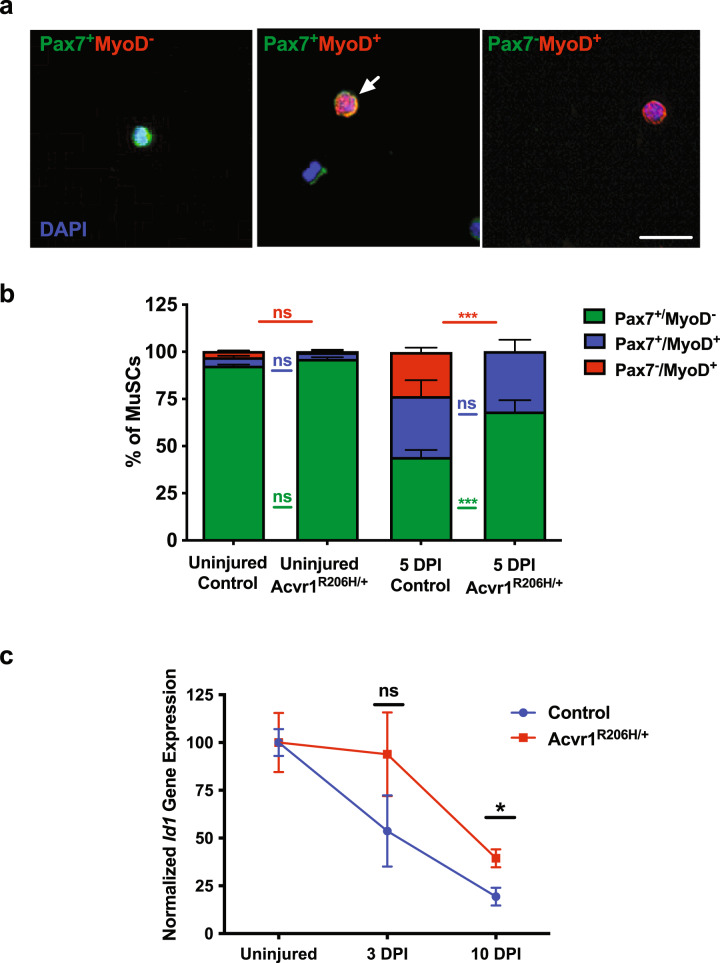
Impaired differentiation of Acvr1^R206H/+^ MuSCs in vivo. **a** Representative images of FACS-isolated MuSCs that were stained for the muscle stem cell marker Pax7 (green), the myogenic commitment marker MyoD (red), and nuclear DAPI (blue); a representative Pax7^+^MyoD^+^ cell is indicated (arrow). Scale bar = 50 µm. **b** Quantification of the percentage of muscle stem cells that are Pax7^+^MyoD^−^ (quiescent; green bar), Pax7^+^MyoD^+^ (early differentiation; blue bar), and Pax7^−^MyoD^+^ (committed differentiation; red bar) from uninjured and injured muscles at 5 DPI. *N* *≥* 3 mice for each group; *N* *>* 100 MuSCs per timepoint and genotype were analyzed. Statistical significance was determined by two-way ANOVA. ***p *<* .0001; ns not significant. Color of statistics bars refers to groups being compared. **c** qRT-PCR expression of Id1 relative to Gapdh expression. *p *<* 0.05. Statistical significance was determined using an unpaired *t*-test. *N* *=* 2–3 mice per genotype per condition.

To further examine myogenic differentiation, we FACS-isolated MuSCs from control and *Acvr1*^*R206H/+*^ mice (uninjured and 5 days post-CTX injury) and examined the expression of the muscle stem cell marker Pax7^28^ and the committed muscle lineage marker, MyoD^30^. Cells were categorized into three groups^31^: Pax7^+^MyoD^−^ (quiescent and/or self-renewing), Pax7^+^MyoD^+^ (early differentiation, myoblasts), and Pax7^−^MyoD^+^ (committed to differentiation) (Fig. 5a). As expected, MuSCs from uninjured control and *Acvr1*^*R206H/+*^ animals were almost exclusively Pax7^+^MyoD^−^ (green) (Fig. 5b, Supplementary Table 1), since MuSCs are quiescent until activated by injury to skeletal muscle tissue^10,24^. At 5 DPI, the proportion of Pax7^+^MyoD^+^ and committed Pax7^−^MyoD^+^ cells in controls (Fig. 5b, Supplementary Table 1) is consistent with the expected myogenic differentiation program during regeneration^24,31^. Interestingly, similar percentages of Pax7^+^MyoD^+^ control and *Acvr1*^*R206H/+*^ expanding Pax7^+^MyoD^+^ myoblasts were observed at 5 DPI, indicating that early differentiation in *Acvr1*^*R206H/+*^ MuSCs is not impaired by the mutation. However, we observed an increased ratio of Pax7^+^/MyoD^−^ undifferentiated cells at 5 days post-CTX injury (Fig. 5b, Supplementary Table 1), indicating that there are elevated MuSC numbers remaining within the regenerating *Acvr1*^*R206H/+*^ tissue for a longer period of time. Additionally, we could not detect *Acvr1*^*R206H/+*^ MuSCs that were committed to differentiation (Pax7^−^MyoD^+^) at 5 DPI, further suggesting delayed myogenic differentiation that maintains early-stage myogenic identity during regeneration. To further confirm that ACVR1 signaling operates as a negative regulator of myogenic differentiation, we isolated MuSCs from control and *Acvr1*^*R206H/+*^ mice and investigated expression of the early differentiation marker myogenin. This analysis demonstrated a reduced percentage of myogenin positive cells in MuSCs isolated from *Acvr1*^*R206H/+*^ mice compared to controls after culturing (Supplementary Fig. 5). Altogether, our data suggest that in the presence of the *Acvr1*^*R206H*^ mutation, a proportion of the MuSC population fails to progress into the myogenic lineage, leading to increased absolute MuSC numbers post-injury, similar to the effect seen in other diseased models with MuSC differentiation inadequacies during regeneration^32–34^. To exclude the possibility that impaired myogenic differentiation is due to aberrant differentiation of MuSCs into non-myogenic HO associated lineages, we investigated the expression of known chondro/osteogenic markers (Runx2, Sox9, Osterix, ALP)^35,36^ by quantitative RT-PCR. These markers, which are highly expressed in chondrogenic ATDC5 cells or the osteoblast precursor cell line MC3T3, were undetected in control or *Acvr1*^*R206H/+*^ MuSCs (Supplementary Fig. 6). MuSCs also did not undergo osteogenic differentiation when exposed to osteogenic culture conditions with or without the addition of BMP4 ligand (Supplementary Fig. 6). These results indicate that trans-differentiation of *Acvr1*^*R206H/+*^ MuSCs to HO precursor cells is unlikely to occur during regeneration, consistent with what has been suggested during development^6,7^.

Inhibitor of differentiation 1 (*Id1*) regulates myogenic differentiation by binding directly to the promoters of myogenic regulatory factors (MRFs: MyoD, myogenin, Myf-5 and MRF4/Myf-6) to prevent their transcription early in myogenesis^37^. Following MuSC proliferative stages, *Id1* must be downregulated to ensure appropriate differentiation during normal regeneration^38^. To further confirm the differentiation impairment of *Acvr1*^*R206H/+*^ MuSCs, we examined *Id1* expression in control and *Acvr1*^*R206H/+*^ MuSCs during post-injury regeneration. Our analysis demonstrates that *Id1* expression is consistently higher in *Acvr1*^*R206H/+*^ MuSCs post-injury (Fig. 5c), in agreement with their reduced differentiation capacity. Taken together, these results further establish an increased propensity for myogenic differentiation defects after injury in *Acvr1*^*R206H/+*^ MuSCs.

### MuSC-specific expression of the FOP mutation (Acvr1^R206H-MuSC^) in vivo does not impact muscle regeneration

**Fig. 6:**
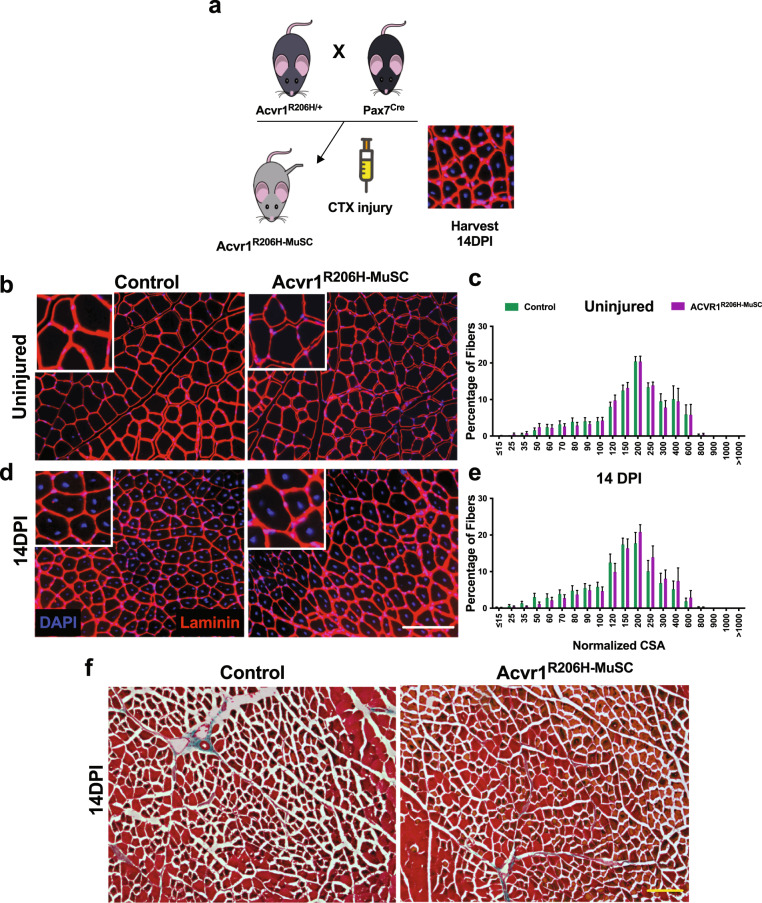
MuSC-specific expression of the FOP mutation (Acvr1^R206H-MuSC^) in vivo does not impact muscle regeneration. **a** Schematic for generation of *Acvr1*^*R206H-MuSC*^ mice and experimental design. Laminin staining of Tibialis Anterior muscle cryosections was conducted on uninjured (**b**) and injured (by 14 DPI) (**d**) control and *Acvr1*^*R206H-MuSC*^ mice. Scale bar = 100 μm. **c, e** Analysis of fiber areas from **b, d**. **f** Trichrome stained cryosections of injured muscle did not detect cartilage or bone in the *Acvr1*^*R206H-MuSC*^ mouse model post-injury and muscle regeneration appears similar to controls. Scale bar = 100 μm. *n* ≥ 4 mice per genotype per treatment condition. Statistical significance was examined by *t*-tests.

To test whether the *Acvr1*^*R206H/+*^ mutation directly alters the function of MuSCs and their progeny in vivo, or whether altered MuSC function requires expression of the mutation in other muscle-resident cell types, we generated mice expressing *ACVR1*^*R206H*^ specifically in MuSCs by crossing the well-established Pax7-Cre line^39^ with floxed *Acvr1*^*R206H/+*^ knock-in mice. The resulting recombined mice (Supplementary Fig. 7), called *Acvr1*^*R206H***-**MuSC^, were injured with CTX intramuscularly along with unrecombined controls and muscle regeneration capacities were analyzed (Fig. 6a). In the absence of injury, *Acvr1*^*R206H-MuSC*^ fibers have similar morphology compared to controls (Fig. 6b) and we detected no difference in fiber area (Fig. 6c), demonstrating that activation of the *ACVR1*^*R206H*^ mutation specifically in MuSCs did not alter muscle morphology. However, after injury we still did not observe any significant difference in sizes of regenerating fibers between *Acvr1*^*R206H-MuSC*^ and control muscles (Fig. 6d, e), indicating proper differentiation of MuSCs. Moreover, muscle regeneration appears to occur comparably to controls and no cartilage or bone was evident in the *Acvr1*^*R206H***-**MuSC^ muscles by 14 DPI (Fig. 6f), suggesting that heterotopic endochondral bone does not form when the mutation is induced solely in MuSCs. Altogether, these genetic data further support that changes in the intrinsic myogenic potential of MuSCs in *Acvr1*^*R206H/+*^ mice are unlikely to account for the decline of their regeneration capability but rather are influenced by the local tissue environment.

### Control FAPs support myogenic differentiation of Acvr1^R206H/+^ MuSCs in vitro

**Fig. 7:**
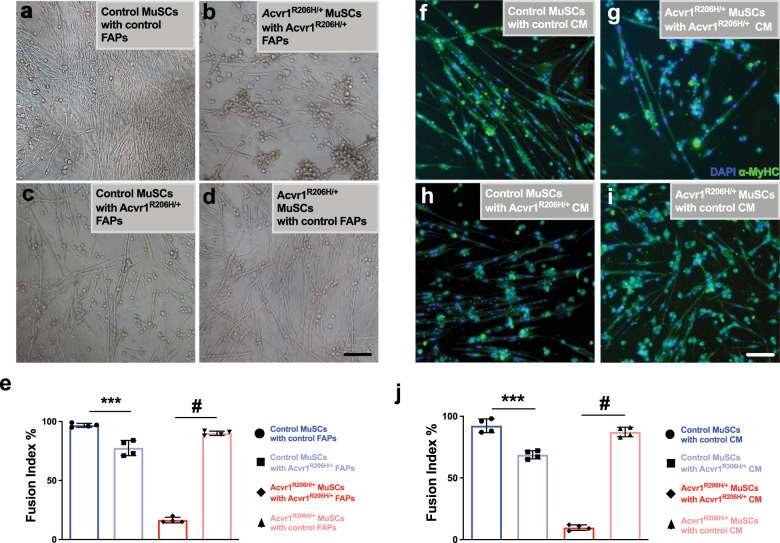
Myogenesis is rescued in Acvr1^R206H/+^ MuSCs cultured with control FAPs. **a–d** Co-cultures of all combinations of mutant and control FAPs and MuSCs were examined. Control MuSCs cultured with Acvr1^R206H/+^ FAPs show reduced efficiency of myofiber formation (**c**), while control FAPs cultured with Acvr1^R206H/+^ MuSCs form mature myofibers that fuse in vitro (**d**) rescuing the myogenic failure of Acvr1^R206H/+^ MuSCs cultured with Acvr1^R206H/+^ FAPs (**b**). **e** Quantification of the fusion index percentage in all co-culture conditions. **f–i** All combinations of mutant and control MuSCs with conditioned media (CM) from mutant and control FAPs were examined. MuSC cultures were stained with the mature muscle marker myosin heavy chain (α-MyHC). *Acvr1*^*R206H/+*^ MuSCs cultured with control FAP-conditioned media for 7 days partially rescued the poor differentiation in Acvr1^R206H/+^ MuSCs (**i**). **j** Quantification of the fusion index percentage in all CM conditions, *n* = 3–4 for each genotype (mean ± SEM). Scale bars for all images = 100 µm. Statistical significance determined by one-way ANOVA, ***p < 0.0002, ^#^*p* < 0.0001.

Previous in vitro studies suggested that the myogenic differentiation of MuSC-derived myoblasts are influenced by FAPs^13^. To assess the specific relationship between FAPs and MuSCs in FOP during myogenic differentiation, we mixed (1:1) and co-cultured FAPs and MuSCs for 7 days under myogenic differentiation conditions. As expected, when control MuSCs were co-cultured with control FAPs, cells differentiated normally, forming elongated branching myofibers (Fig. 7a), consistent with previous findings^13^. However, *Acvr1*^*R206H/+*^ MuSCs cultured with *Acvr1*^*R206H/+*^ FAPs showed minimal or no fusion, and the few myofibers observed had no branches and decreased fusogenic ability (Fig. 7b). Interestingly, when *Acvr1*^*R206H/+*^ FAPs were co-cultured with control MuSCs, the control MuSCs did not fuse efficiently, forming shorter and wider fibers (Fig. 7c, e) as compared to control MuSC-FAP co-cultures. This result demonstrates that the FOP mutation in FAPs negatively influences the myogenic potential of MuSCs. FAP influence on MuSCs was further demonstrated when mutant MuSCs were co-cultured with control FAPs: the control FAPs ameliorated the myogenic morphology of *Acvr1*^*R206H/+*^ MuSCs, resulting in increased fusion of MuSCs and formation of elongated branching fibers (Fig. 7d, e). These data highlight the ability of healthy FAPs to influence and support proper cell differentiation of FOP MuSCs.

To investigate whether MuSCs and FAPs require direct contact for proper differentiation or whether FAPs influence MuSCs through secreted factors that promote myogenesis, we generated conditioned media (CM) by culturing FAPs in media separately and independently of MuSCs. We collected CM from the FAPs and cultured MuSCs in FAP CM for one week. Control MuSCs cultured with control FAP CM retained normal differentiation potential (Fig. 7f, j), while *Acvr1*^*R206H/+*^ MuSCs cultured with *Acvr1*^*R206H/+*^ FAP CM showed impaired differentiation (Fig. 7g, j). Control MuSCs cultured with *Acvr1*^*R206H/+*^ FAP CM were unable to fuse and form myofibers as proficiently as control MuSCs with control FAP CM (Fig. 7h, j). Interestingly, *Acvr1*^*R206H/+*^ MuSCs cultured with control FAP CM had reduced pSmad1/5/8 levels (Supplementary Fig. 8), were rescued from impaired differentiation and attained fusion indices similar to control MuSCs (Fig. 7i, j), consistent with the cell co-culture experiments (Fig. 7a-e). Taken together, these data demonstrate an impaired ability of *Acvr1*^*R206H/+*^ FAPs to support MuSC myogenic potential in FOP. To further address the ACVR1 signaling in MuSCs with conditioned media from control and *Acvr1*^*R2006H/+*^ FAPs, we investigated the expression of several TGFβ/BMP pathway component proteins in FAP-conditioned media from cells isolated from control and *Acvr1*^*R206H/+*^ mice, using a commercially available array. We found several dysregulated proteins (Supplementary Fig. 9), such as upregulation of the BMP2 ligand and downregulation of the BMP antagonist Noggin in CM of *Acvr1*^*R206H/+*^ FAPs, consistent with previous reports correlating similar changes with impaired myogenic differentiation of MuSCs^40^. Collectively, our data indicate that muscle regeneration is impaired in FOP skeletal muscle tissue due to the altered function of FAPs and their impact on the myogenic differentiation of MuSCs.

## Discussion

The replacement of skeletal muscle tissue and other soft connective tissues with bone tissue in FOP in response to injury indicates that muscle regeneration mechanisms that normally maintain and/or repair muscle tissue are severely perturbed. Previous work has examined the origins of heterotopic ossification (HO)^6,7^ and the effect of BMP in muscle atrophy^41,42^, but the impact of *ACVR1*^*R206H*^ on muscle tissue regeneration remained unclear. In this study, we demonstrate that accompanying the development of HO, *Acvr1*^*R206H/+*^ muscle tissue regenerates poorly after injury. Utilizing a knock-in *Acvr1*^*R206H/+*^ mouse model^43^, we show that under steady-state conditions, *Acvr1*^*R206H/+*^ *and* control skeletal muscle are morphologically similar. However, 5 days following injury, regenerating fibers in the *Acvr1*^*R206H/+*^ muscles were smaller compared to the size of the centralized nuclei fibers seen in control regenerating muscles, signifying a deficiency in the regenerative capacity of FOP muscles. Thus, in addition to the formation of HO, the *ACVR1*^*R206H*^ mutation is responsible for inefficient regeneration of the skeletal muscle tissue after injury.

Muscle injury stimulates MuSCs to exit quiescence and begin proliferation^24^. Within a few days after injury, MuSCs switch from a proliferative stage to a differentiation stage. Eventually, committed muscle cells fuse to regenerate muscle fibers to restore damaged muscles^10,24^. At the same time as MuSC activation, FAPs also begin to proliferate in response to injury to facilitate myogenesis^13^. In the present study, we investigated the kinetics of *Acvr1*^*R206H/+*^ MuSCs and FAPs after CTX injury and found that their proliferation capacities are comparable to controls, suggesting that the initial response of *Acvr1*^*R206H/+*^ skeletal muscle to injury is normal. The similar rates of MuSC proliferation correlate well with the similar morphologies of *Acvr1*^*R206H/+*^ and control muscles during the first days after injury. Therefore, the reduced size of centralized (newly forming) myofibers in *Acvr1*^*R206H/+*^ muscles cannot be attributed to proliferation defects of muscle-resident progenitors. We also excluded other possible etiologies, such as MuSC loss due to increased cell death or MuSC trans-differentiation to chondrogenic/osteogenic lineages. Since loss of regenerative capacity was not due to proliferation defects, stem cell loss or fate alteration, we reasoned that the mutation of the BMP type I receptor ACVR1 in FOP might antagonize cell differentiation. We confirmed this hypothesis by finding decreased numbers of committed MuSCs and a concomitant increased ratio of Pax7^+^/MyoD^−^ undifferentiated cells in *Acvr1*^*R206H/+*^ muscle at 5 days post-injury as well as decreased myogenin expression in MuSC-derived myoblasts. We further determined that the delayed myogenesis in injured *Acvr1*^*R206H/+*^ skeletal muscle tissue is due, at least in part, to decreased expression of the mature myogenic marker α-MyHC as disruption of myogenic maturation after injury has been reported to impact the outcome of muscle regeneration^11^. Interestingly, we determined that *Id1* expression is increased in *Acvr1*^*R206H/+*^ MuSCs. These results are in agreement with previous reports suggesting that elevated *Id1* expression can participate in the inhibition of myogenesis^40^.

Myogenesis and muscle differentiation are supported by FAPs, which provide a favorable environment promoting MuSC-mediated regeneration^13,16,44,45^. Muscle injury stimulates FAPs to produce paracrine factors that promote MuSC-mediated homeostasis^13,46^, in part by disrupting the environment conducive for muscle regeneration^18,27^. Around 3 days post-injury, FAPs typically reach their peak proliferation and then quickly recede to pre-injury levels by apoptosis^13,17,19^. In contrast, in muscles from *Acvr1*^*R206H/+*^ mice, we found decreased apoptosis and higher FAP numbers at later stages of regeneration, revealing a defect in the mechanism of FAP clearance. It is possible that the prolonged persistence of diseased FAPs within the regenerating muscle contributes to the altered muscle environment in FOP, implicating FAPs as the predominant cell-of-origin for the formation of HO and in agreement with a previous study^6^. However, the effects of MuSC-FAP communication during myogenesis in adult FOP muscle regeneration remained unexplored, a gap of information that precludes effective strategies towards promoting muscle regeneration at the expense of heterotopic bone formation. Here, we first examined the intrinsic differentiation potential of these cell types in vitro. *Acvr1*^*R206H/+*^ MuSCs cultured with *Acvr1*^*R206H/+*^ FAPs resulted in stunted MuSC differentiation. Surprisingly, *Acvr1*^*R206H/+*^ FAPs negatively impacted the differentiation ability of control MuSCs in co-culture experiments, demonstrating that changes in FAP activity contribute to the myogenic behavior of MuSCs. Indeed, when *Acvr1*^*R206H/+*^ MuSCs were cultured in the presence of control FAPs, they exhibited somewhat improved differentiation, indicating that FAP activity dominates the intrinsic myogenic commitment of MuSCs. Importantly, MuSC-specific activation of the *ACVR1*^*R206H*^ mutation in vivo was not sufficient to impair regeneration, further supporting that FAPs communicate with FOP MuSCs and provide a permissive muscle tissue environment that promotes disease progression. Collectively, these results indicate that the progressive failure of MuSCs to support regeneration in *Acvr1*^*R206H/+*^ muscles is likely determined by extrinsic alterations in the muscle tissue environment that compromise productive interactions between FAPs and MuSCs. In support of this idea, control FAP-conditioned media was shown to reduce the pSmad1/5/8 levels and potentiate the ability of mutant MuSCs to form multinucleated myotubes, mirroring the results of the co-culture experiments. These results support our hypothesis that *Acvr1*^*R206H/+*^ FAPs are modulating the myogenic program, at least partially through the secretion of soluble factor(s). Our TGFβ/BMP pathway signaling proteins analysis showed elevated BMP secretion by *Acvr1*^*R206H/+*^ FAPs, as well as downregulation of BMP antagonists. These findings further support the role of *Acvr1*^*R206H/+*^ FAPs in modulating FOP myogenic differentiation. Nevertheless, these data cannot determine whether these are primary or secondary molecular changes or whether the myogenic responses seen in *Acvr1*^*R206H/+*^ MuSCs are influenced by other factor(s) secreted earlier from *Acvr1*^*R206H/+*^ FAPs that affected the above molecules or the result of secretion of an inhibitory factor(s) against myogenesis. Future studies will be necessary, including mouse models that genetically target candidates specifically in FAPs, to define the primary key molecular determinants produced by *Acvr1*^*R206H/+*^ FAPs that dictate MuSC dysfunction and promote a tissue environment that suppresses muscle regeneration and is conducive for osteogenesis in FOP.

**Fig. 8:**
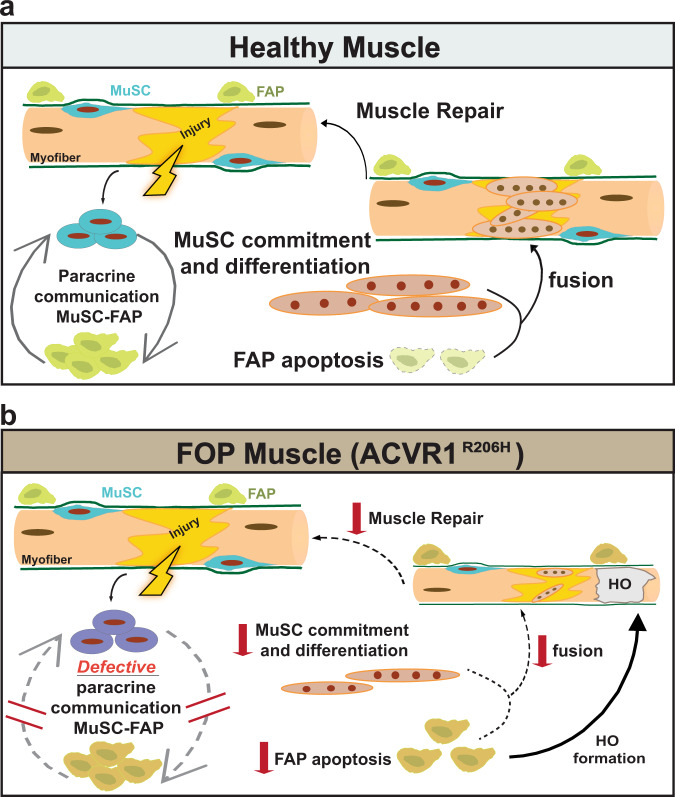
Schematic Illustration of the proposed model for muscle regeneration in healthy and FOP muscles. **a** In healthy muscles, injury activates MuSCs and FAPs to expand. FAPs sustain MuSC-mediated commitment and differentiation (myotubes) by providing transient paracrine signals at the sites of damage. Finally, FAP numbers decline by apoptosis to pre-injured numbers, while myotubes fuse to repair damaged myofibers. **b** In FOP (*ACVR1*^*R206H*^) muscles, activation and expansion of MuSCs and FAPs is normal. However, the defective paracrine communication between MuSCs-FAPs, reduces the myogenic abilities of MuSCs and skeletal muscle repair is diminished. At the same time, diseased FAPs fail to decline by apoptosis and contribute to bone formation within FOP regenerating fibers.

In summary, our data highlight the detrimental effect of the *Acvr1*^*R206H/+*^ FAPs on regulating MuSC performance to repair skeletal muscles. In healthy muscles, upon injury MuSCs-FAPs paracrine communication supports the regenerative dynamics of MuSCs, resulting in the reduction of FAPs by apoptosis and in MuSC commitment/differentiation at the later stages of regeneration (Fig. 8a). In contrast, we propose that under the influence of the mutation, the defective reciprocal interaction between MuSCs-FAPs in diseased muscles, together with the persistent abundance of FOP FAPs, reduces the myogenic capabilities of MuSCs and skeletal muscle repair is diminished. At the same time, imposed by the excess of *Acvr1*^*R206H/+*^ FAPs, extraskeletal bone (HO) begins to form within regenerating fibers (Fig. 8b). It is likely that the reduced myogenic differentiation potential of the *Acvr1*^*R206H/+*^ MuSCs further exacerbates the already challenged muscle regeneration of the tissue, where during an attempt to repair muscle, HO occupies the damaged area. We propose that therapeutic interventions should consider boosting the myogenic potential of regenerating muscles, along with reducing ectopic bone formation. The data presented here document that FOP is not only a disease of heterotopic ossification development but also a breakdown of canonical skeletal muscle tissue function and muscle regeneration. Taken together, this study uncovers a previously unrecognized role of the *ACVR1*^*R206H*^ mutation on MuSC-FAP interaction during the progression of FOP and provides the foundation for targeting this interrelationship in future therapeutic approaches in order to improve muscle repair.

## Methods

### Mice

A conditional-on knock-in mouse Acvr1^[R206H]FlEx/+^^43^ was used to generate mice with doxycycline-inducible global allele expression: Acvr1^[R206H]FlEx/+^;Gt(ROSA) 26Sor^tm1(rtTA*M2)Jae^; Tg(tetO-Cre)1Jaw mice (referred to as *Acvr1*^*R206H/+*^ in the manuscript). To induce recombination and global expression of the mutant allele, 4-week-old mice were provided doxycycline chow (625 mg/kg, Envigo RMS Inc., TD 01306) for five consecutive days. Cre-recombination was verified by PCR. Littermate *Acvr1*^*+/+*^ controls were also treated with doxycycline chow. For MuSC-specific expression, Pax7-Cre mice^39^ (JAX #010530) were crossed to the Acvr1^[R206H]FlEx/+^ mouse. All mice were maintained under 12 h light/12 h dark cycles with unlimited access to food and water. All animal experiments were conducted in compliance with the regulations of University of Pennsylvania and were approved by the Institution’s Animal Care Committee.

### Skeletal muscle injury

Acute skeletal muscle injuries were performed as previously described^9^. More specifically, Tibialis Anterior and Gastrocnemius muscles of 2-month-old mice were injected intramuscularly with 10 or 20 µL, respectively, of 20 µM cardiotoxin (CTX) from *Naja mossambica mossambica* (Sigma–Aldrich, St. Louis, MO; C9759). Muscle tissue was collected at 0-, 3, 5-,7, 10-, and 21-days post-injury (DPI).

### MuSC and FAP isolation

MuSC isolation was performed as described^47^. Briefly, hindlimb muscles were dissected from 2-month-old mice. Muscle was finely minced and enzymatically digested with collagenase and dispase. Single-cell suspensions were generated by passing cells through a 21-gauge needle and filtered through a 40 µM cell strainer. Cells were incubated with 1× red cell lysis buffer (eBioscience) and biotinylated antibodies against CD45, CD31, CD11b, and Sca1 (Supplementary Table 2). Cells were centrifuged and further detected for antibodies against CD34, and α7- integrin, as well as streptavidin-PE-Cy7 (Supplementary Table 2). Prior to FACS sorting, the viability dye 7-aminoactinomycin D (7-AAD; Sigma–Aldrich) was added (final concentration 4 µg/mL). MuSCs were identified as CD45−/CD31−/CD11b−/Sca1−/ α7-integrin + /CD34 + and were sorted using a BD Aria II. Information about lasers and filter sets can be found in Supplementary Table 3. FAPs were collected as CD45−/CD31−/CD11b−/α7-integrin-/Sca1 + , with or without CD34 + staining. MuSCs were sorted into 1.5 mL microcentrifuge tubes containing 500 µL of cold myoblast media (DMEM/Ham’s F12; 15% FBS; 1× NEAA (Gibco), 1× GlutaMAX (Gibco), 1× anti-anti (Fisher Scientific)). FAPs were sorted into 1.5 mL microcentrifuge tubes containing 500 µL of cold FAP media (DMEM; 20% FBS; 1× anti-anti). The collection tubes were maintained at 4 °C during the sorting process using a circulating water system. Data were collected from 20,000 total events and analyzed using FlowJo 10.1 software.

### Proliferation assay

To assess the proliferation of MuSCs and FAPs, 2.5 mg BrdU (Roche, Basel, Switzerland) was injected intraperitoneally (100 mg/kg) in PBS with a 29-gauge insulin syringe 24 h before cell isolation. For detection of BrdU, isolated cells were plated on collagen-coated six-well chamber slides at 37 °C overnight, following FACS isolation. Cells were briefly washed with PBS and processed with the 5-Bromo-2’-deoxy-uridine Labeling and Detection Kit I (Roche), according to the manufacturer’s instructions. Briefly, cells were fixed [70% ethanol, 30% 50 mM glycine (pH 2, Santa Cruz)] for 20 min at room temperature, washed for 10 min with PBS, and blocked with blocking buffer (20% goat serum/0.3% Triton-X in PBS) for 1 h at room temperature. Cells were stained with anti-BrDU antibody provided with the kit in Incubation buffer (1:10; Roche) for 30 min at 37 °C. After washing with PBS, Alexa Fluor 488-conjugated goat anti-mouse IgG secondary antibody (1:1000 in blocking buffer) was added and incubated in the dark for 1 h at room temperature. Cells were washed, chambers removed, and coverslips were mounted with fluoromount G plus DAPI (SouthernBiotech, Birmingham, AL). Cells stained for BrdU were imaged using an Eclipse TE2000-U inverted fluorescent microscope (Nikon, Minato City, Tokyo, Japan) and BrdU-positive cells were quantified in Image J.

### Differentiation assay

FACS-isolated MuSCs were grown on collagen-coated (C8919, Sigma) six-well chamber slides (2000/well, Lab-Tek) and maintained in myoblast growth media composed of Ham’s F-10 (Gibco) and 15% FBS (Omega Scientific) with 2.5 ng/ml hFGF (G5071, Promega, Madison, WI). Cells were expanded and passaged by dissociation with Accumax (Millipore, Burlington, MA). When confluent, cells were cultured in low serum myogenic differentiation media (DM) composed of DMEM with 5% HS (horse serum; 16050-122, Gibco). Following 7 days of culture in DM, cells were fixed with 4% paraformaldehyde/PBS for 20 min and washed once with PBS. Cells were permeabilized with 0.5% Triton X-100/PBS for 30 min, washed twice with PBS, and blocked for 1 h at room temperature in 3% BSA/PBS. Cells were stained for α-MyHC (Developmental Studies Hybridoma Bank/DSHB, 1/10 in 3% BSA/PBS) overnight at 4 °C, washed with PBS, and stained with Alexa Fluor 488-conjugated goat anti-mouse IgG (Life Technologies; 1/500 in 3% BSA/PBS) at room temperature for 1 h. Cells were washed, chambers removed, and coverslips were mounted with fluoromount G plus DAPI (SouthernBiotech). Cells were imaged using an Axio observer microscope (Zeiss, Oberkochen, Germany). The fusion index was calculated as the number of nuclei in multinucleated myotubes divided by the total number of nuclei (as in ref. ^48^). For myogenin staining, MuSCs were grown as above and placed in DM medium for 1 day before staining and imaging. Myogenin antibody (DSHB, clone F5D, 1/10 dilution) primary antibody was used, along with Alexa Fluor 647-conjugated goat anti-mouse secondary antibody (Thermo; 1/500).

### MuSC immunofluorescence staining

FACS-sorted MuSCs from uninjured and injured mice were cytospun onto laminin (Roche)-coated *Superfrost Plus* microscope slides (Thermo Fisher Scientific, Waltham, MA). Cells were fixed for 10 min in 4% PFA and rinsed with PBS, then permeabilized with 0.5% Triton-X for 15 min, washed with PBS, and blocked with Avidin Biotin kit (Vector Laboratories, Burlingame, CA) per kit instructions. Cells were subsequently blocked with M.O.M (Mouse on Mouse) blocking reagent kit (Vector Laboratories) for 1 h at 37 °C, followed by another blocking period with 3%BSA/0.1% Triton-X/PBS for 1 h at room temperature. Cells were incubated overnight with primary antibodies for Pax7 (Thermo, 1:50) and MyoD (DSHB, 1:250) at 37 °C, overnight, then washed with PBS and blocked with the mouse biotinylated reagent from the M.O.M kit (Vector Laboratories) in 3% BSA/0.1% Triton-X/PBS for 30 min at room temperature. Cells were then incubated with goat anti-rabbit Alexa Fluor 647 (1:250, Sigma) and Alexa Fluor 488 streptavidin (1:250, Biolegend, San Dirego, CA) in 3% BSA/0.1% Triton-X/PBS for 1 h at room temperature. Slides were mounted with Prolong Gold (Thermo) and stained with DAPI. Cells were imaged with an Eclipse 90i microscope (Nikon) at consistent exposure times. Cells were analyzed for expression of Pax7/MyoD/DAPI via Image J. For staining of FAPs and MuSCs for pSmad1/5/8, FACS-isolated MuSCs and FAPs were grown on collagen-coated eight-well chamber slides (Thermo). Before harvest, cells were washed twice with PBS and incubated for 7 h in serum-free DMEM containing 30 ng/mL BMP4 (R&D Systems). In the case of conditioned media, cells were washed with PBS and incubated with FAP-conditioned media from the indicated genotypes. In both cases, cells were then fixed in 4% PFA/PBS for 10 min, permeabilized with 0.5% Triton X-100/PBS for 5 min, and blocked with 3% BSA/0.1% Triton X-100/PBS for 1 h. Phospho-Smad1/5/8 antibody (1:200; Cell Signaling Technology) was incubated overnight in blocking buffer at 4 °C. Cells were washed with PBS and stained with goat anti-rabbit Alexa Fluor 555 secondary antibody (1/500, Thermo) in blocking buffer for 1 h at room temperature. After PBS washing, chambers were removed and coverslips were mounted with Fluoromount G with DAPI (Southern Biotech). Slides were imaged on an Eclipse 90i with the same exposure settings between groups.

### Culture conditions of FAPs and MuSCs

Both MuSCs and FAPs were grown on collagen-coated (C8919, Sigma) six-well chamber slides (company) and maintained in growth media composed of Ham’s F-10 (Gibco) or DMEM (Gibco) and 15% and 20% FBS, respectively (Omega Scientific) with 10 mg/ml hFGF (G5071, Promega). Both were expanded and passaged by dissociation with Accumax (Millipore). Once cells were confluent, they were cultured with myogenic differentiation media composed of DMEM with 5% HS (horse serum; 16050-122, Gibco) for 14 days then stained with Hoechst and detected for α-MyHC (DSHB; 1/10 in 3% BSA/PBS). For co-culture experiments, MuSCs and FAPs were seeded 1:1 (2,000 cells/well each cell type) and cultured in myogenic differentiation media for 7 days, with the media replenished every other day. For conditioned media experiments, FAPs were cultured in FAP media for 7 days, which was then collected, and MuSCs were then cultured in FAP-conditioned media for a week. Each experiment was performed three times with *n* = 3–4 mice per condition each time.

### Micro-computed topography analysis

21 days following injury, mice were euthanized and both lower hind limbs were collected and fixed for 2 h in 4% paraformaldehyde at 4 °C. HO was detected and quantified in high‐resolution, cross‐sectional reconstructed images of paraformaldehyde (PFA)‐fixed hind limbs using micro-computed tomography (μCT) VivaCT40 imager (Scanco Medical AG, Brüttisellen, Switzerland) at a source voltage of 55 kV, a source current of 145 µA, and an isotropic voxel size of 19.0 µm. Three‐dimensional renderings to quantify HO were reconstructed using Scanco μCT V6.1 software from regions of interest that were free‐hand drawn around HO every 5–10 reconstructed slices and then interpolated for total volume. Users ensured that the HO region of interest did not include skeletal bone. Thresholding values for HO detection ranged from 240 to 1000 Hounsfield units.

### Muscle morphology

Tibialis Anterior muscles were collected at timepoints after injury (0, 5, 10, and 21 days), fixed in 4% PFA for 2 h with rocking at 4 °C, and decalcified in 10% EDTA (pH 7) for 72 h. TAs were then suspended in 30% sucrose overnight and embedded in Optimal Cutting Temperature (O.C.T.) Compound (Sakura Finetek USA, Torrance, CA) the next morning. Cryosections (10 µm slices) from *Acvr1*^*+/+*^ and *Acvr1*^*R206H/+*^ mice were cut onto *Superfrost Plus* slides. Slides were stained with standard hematoxylin and eosin (H&E) to visualize gross tissue morphology. Alcian blue hematoxylin/orange G stain was used to visualize HO within the skeletal muscle as described in ref. ^49^. Muscle tissue sections were imaged using an Eclipse 90i microscope (Nikon). Centralized nuclei myofibers were quantified in Image J from the H&E stained sections of *n* = 3 individual mice per genotype and per condition. Cross-sectional area values from each mouse were normalized to individual mouse body weight. The investigators were blinded during image analysis.

### Tissue culture and assessment of osteogenic potential

MuSCs were cultured to confluence in myoblast growth media prior to transfer to osteogenic media (10% FBS, 50 μg/ml ascorbic acid, 10 mM β-glycerophosphate (Sigma) in high glucose DMEM as in ref. ^50^), with or without the addition of BMP4 ligand (100 ng/ml) as indicated. Media was replenished every 3 days during the assay. After 14 days, wells were fixed with 4% paraformaldehyde and imaged for cell morphology. Murine mesenchymal progenitor cells were utilized as a positive control for osteogenic differentiation. Cells were cultured in osteogenic media with BMP4 ligand (100 ng/ml) for 14 days and were stained for Alizarin Red following culture to visualize mineralization (as in ref. ^50^).

### TUNEL assay

To assess for the level of MuSC and FAP death following injury, hindlimb muscles of age-matched control and FOP mice were injured with CTX. Tibialis anterior muscles were collected at the indicated timepoints and fixed in cold 4% paraformaldehyde in PBS for 2 h, followed by incubation with 30% sucrose overnight at 4 °C. Muscles were embedded in OCT and frozen in chilled isopentane, and 10 μm sections were placed on *Superfrost Plus* slides, encompassing the entirety of the length of the muscle. Sections were permeabilized with 0.5% Triton X-100/PBS for 15 min, rinsed with PBS, and for MuSC staining, underwent heat-mediated antigen retrieval with 1 mM EDTA, pH8.0/0.05% Tween-20, and stained for DNA fragmentation using a TUNEL using the Click-iT Plus TUNEL Assay for In Situ Apoptosis Detection, Alexa Fluor 647 kit, according to the manufacturer’s instructions with the exception that the proteinase K treatment step was eliminated. After TUNEL staining, sections were stained for MuSC or FAP markers. For MuSCs, sections were first blocked with an avidin/biotin blocking kit (Vector Labs), blocked with a mouse-on-mouse kit (Vector labs), and blocked with a blocking buffer (3% BSA/PBS/0.1% Triton X-100). Antibody against Pax7 (1/20; Santa Cruz) was incubated overnight in blocking buffer at 4 °C. the following day, Pax7 signal was amplified using the mouse-on-mouse kit and sections were incubated in blocking buffer with Alexa Fluor 488-conjugated streptavidin (1/100; Biolegend). For staining FAPs, cryosections were stained with Alexa fluor 488-conjugated PDGFRα antibody (1/100; Santa Cruz). Coverslips were mounted with prolong gold with DAPI (Invitrogen). Sections were imaged on a Nikon eclipse Ni-U equipped with a Nikon Qi1Mc 14-bit camera. MuSC death was determined as DAPI^+^/Pax7^+^/TUNEL^+^, and FAP death was measured as DAPI^+^/PDGFRα^+^/TUNEL^+^, excluding regions near blood vessels.

### Laminin staining and fiber area analysis

TA cryosections (10 µm) prepared as above were permeabilized in 0.5% Triton X-100/PBS for 5 min, blocked with 3% BSA/PBS/0.1% Triton X-100/PBS and stained with antibody to Laminin B2 (1/200; clone A5, Fisher Scientific). Sections were washed with PBS and incubated with goat anti-rat Alexa Fluor 647 (1/400; Invitrogen) before mounting coverslips with prolong gold with DAPI (Invitrogen). Sections were imaged on a Nikon eclipse Ni-U equipped with a Nikon Qi1Mc 14-bit camera. Fiber areas were manually assessed in Fiji/ImageJ.

### RNA Isolation, cDNA synthesis, and quantitative real-time PCR

RNA was isolated from FACS-isolated MuSCs and cultured MC3T3 and ATCD5 cells (cell lines were both gifts from Ling Qin laboratory at the University of Pennsylvania) using the RNeasy Micro Plus Kit (Qiagen, Hilden, Germany). cDNA was synthesized using the Protoscript II first-strand cDNA synthesis kit (New England Biolabs). QPCR was carried out using FAM-conjugated ID1 primer (Assay ID: Mm00775963_g1) Runx2 (Assay ID: Mm00501584_m1), Sox9 (Assay ID: Mm00448840_m1), Osx (Assay ID: Mm04209856), ALP (Assay ID: Mm00475834_m1), and VIC-labeled mouse GAPD (GAPDH) endogenous control (all Applied Biosystems, Foster City, CA). Reactions were amplified on a Quantstudio 6 Real Time PCR instrument (Applied Biosystems) and carried out in triplicate with cells isolated from at least three mice per condition. Data were analyzed using the ΔΔCT method.

### FAP-conditioned medium secreted protein array

Control and *Acvr1*^*R206H/+*^ FAPs were FACS-isolated and plated on 24 well plates. After growth, cells were washed and placed in DMEM with 0.2% serum for 8 h. Conditioned medium (CM) was collected from 4 primary FAP cultures per genotype and pooled, aliquoted into 1 mL aliquots, and stored at −80 °C until processed. On the day of processing, CM was thawed and protein was quantitated with the DC assay (Bio-Rad). A commercially available array specific for BMP and TGFβ targets (Ray Biotech) was used according to the manufacturer’s instructions with the following modifications: Arrays were incubated overnight with conditioned media at 4 °C; arrays then were incubated with biotinylated antibody incubation for 2 h, and finally Streptavidin HRP for 2 h. ECL-incubated membranes were imaged on a G:BOX imaging system with accompanying Chemi-XX6 GENESys software (Syngene).

### Recombination genotyping

Genomic DNA was isolated from hindlimb muscles of 2-week-old pups. For PCR, 1 µg of DNA, 1.25 µL of a 10 µM primer mixture (primer 1: 5’- TGTATTGCAGGACGCTGAAG-3’; primer 2: 5’-CCCCTGAAGTGGAATAACCA-3’), 10.25 µL water, and 12.5 µL of 2X KAPA2G Fast HotStart Genotyping Mix with dye (Kappa Biosystems) was used for amplification. PCR products were electrophoresed on a 3% TAE agarose gel stained with GelRed Nucleic Acid Gel Stain (Biotium) were visualized with a Gel Doc XR + imaging system with Image Lab 5.1 Software (BioRad). A wild-type or unrecombined Acvr1 allele produces a fragment size of ~325 bp, while a recombined allele produces a ~375 bp product.

### Statistical analysis

Data were analyzed statistically using GraphPad (La Jolla, CA) Prism 7 software. Results are presented as the mean ± SD or SEM. Datasets were analyzed using two-tailed, unpaired, Student’s *t*-test or one-way ANOVA (Tukey’s multiple comparison post-hoc test) to determine significance. Differences were considered statistically significant at *p* < 0.05. Significance and sample size are indicated for each dataset in the figure legends.

### Reporting summary

Further information on research design is available in the Nature Research Reporting Summary linked to this article.

## Supplementary information

Supplemental Materials

Reporting Summary

## Data Availability

All data generated during and/or analyzed during this study are included in this published article and its supplementary information. Any detailed data supporting the findings of this study are available from the corresponding authors upon reasonable request.
