# Supplementary material for: Dynamics of skeletal muscle-resident stem cells during myogenesis in fibrodysplasia ossificans progressiva
Source: NPJ Regen Med. 2022 Jan 14;7:5. doi: 10.1038/s41536-021-00201-8 (PMC8760285; doi:10.1038/s41536-021-00201-8)
Supplement: Supplementary file 1 — Supplemental Materials [file 41536_2021_201_MOESM1_ESM.pdf]

Dynamics of skeletal muscle-resident stem cells during myogenesis in fibrodysplasia ossificans progressiva

Alexandra Stanley, Elisia D. Tichy, Jacob Kocan, Douglas W. Roberts, Eileen M. Shore and Foteini Mourkioti

Supplementary Figures and Tables

Supplementary Figures

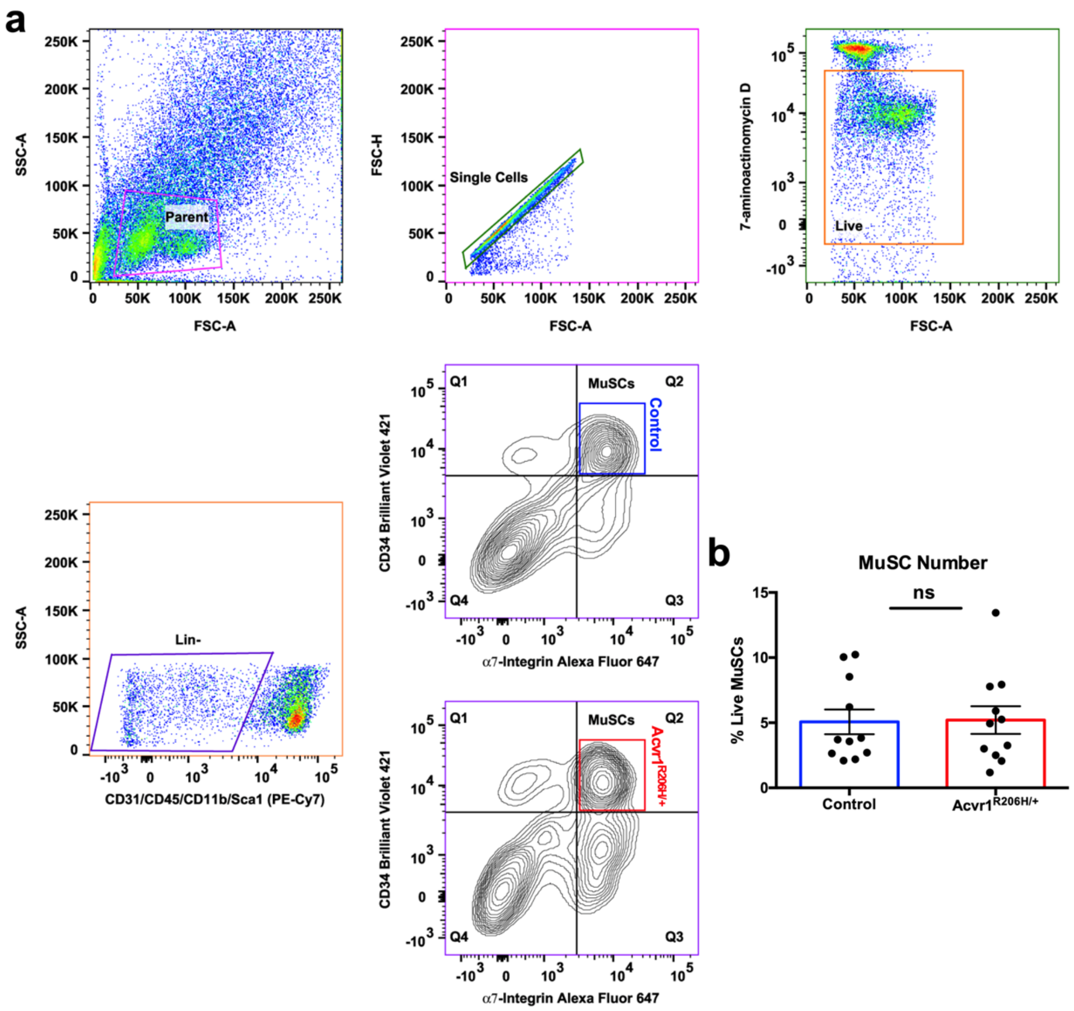

Supplementary Figure 1. Schematic of MuSC isolation from control and *Acvr1*$^{R206H/+}$ skeletal muscles.

a) MuSC isolation by fluorescent-activated cell sorting (FACS). Top: gating strategy of parent population (pink box) of muscle cell isolation, gating of single cells (green box), and selection of live cells (7-AAD negative, orange box). Lower: MuSC enrichment by gating CD11b^{-}/CD45^{-}/CD31^{-}/Sca1^{-} (lineage negative, purple box) populations followed by gating for double positives (CD34^{+}/α7-integrin^{+}). Representative plots of control MuSCs (blue box) and *Acvr1*$^{R206H/+}$ (red box) are shown. **b)** Quantification of number of the percent of live MuSCs isolated from skeletal muscle from control and *Acvr1*$^{R206H/+}$ mice. Graph represents mean ± SEM. n = 10-11 mice for each genotype. Statistical significance by unpaired t-test; ns = non-significant.

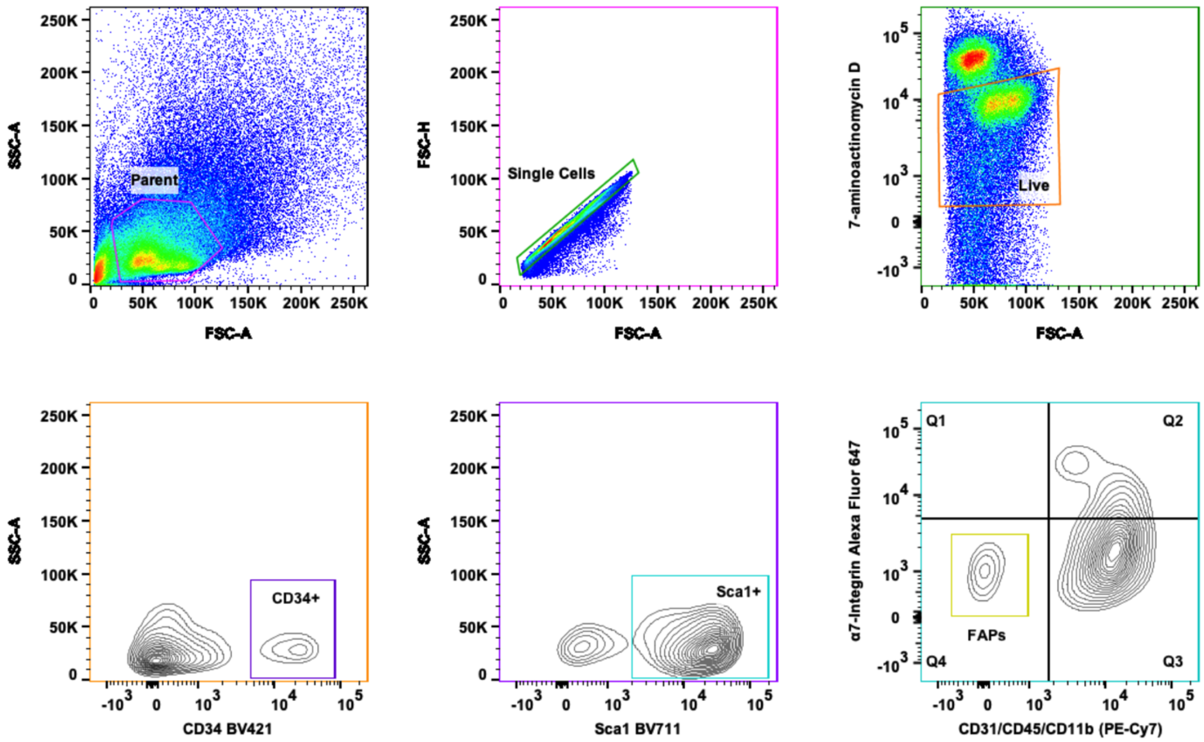

Supplementary Figure 2. Schematic of FAP isolation from skeletal muscles.

FAP isolation by fluorescent-activated cell sorting (FACS). Top: gating strategy of parent population (pink box) of muscle cell isolation, gating of single cells (green box), and selection of live cells (7-AAD negative, orange box). Lower: FAP enrichment by gating CD34+ cells (purple box) followed by separation of the Sca1+ population (aqua box). Lastly, FAPs are identified based on α7-integrin-/CD31-/CD45-/CD11b- populations (yellow box).

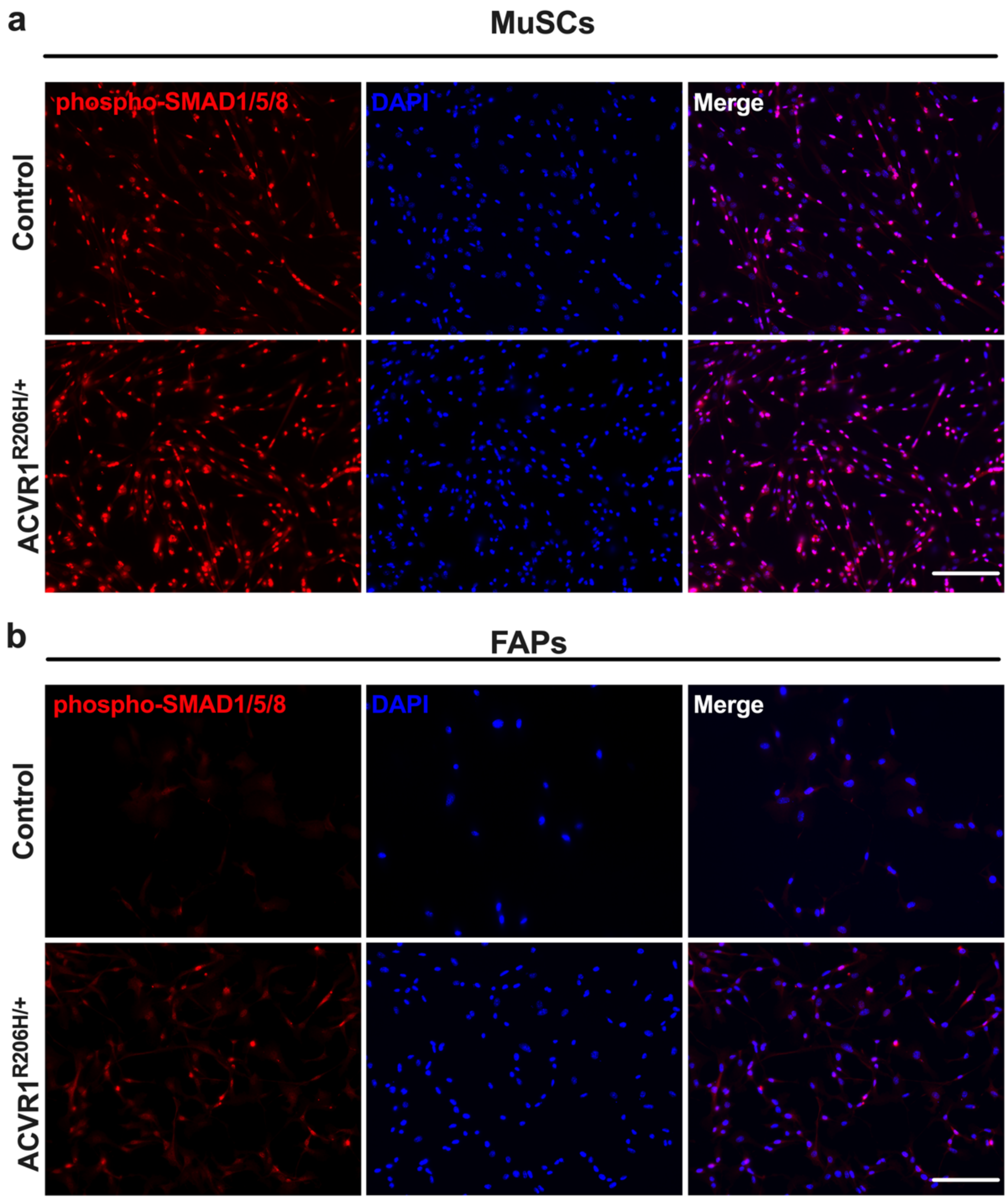

Supplementary Figure 3. Elevated pSmad1/5/8 in both MuSCs and fibroadipogenic progenitors isolated from *Acvr1$^{R206H/+}$* muscles.

a) Representative images of control and *Acvr1$^{R206H/+}$* MuSCs stained with the pSmad1/5/8 antibody (red) and DAPI (nuclei; blue). Scale bar = 100 µm. **b)** Representative images of control and *Acvr1$^{R206H/+}$* FAPs stained with a pSmad1/5/8 antibody (red) and DAPI (nuclei; blue). Scale bar = 100 µm.

a

b

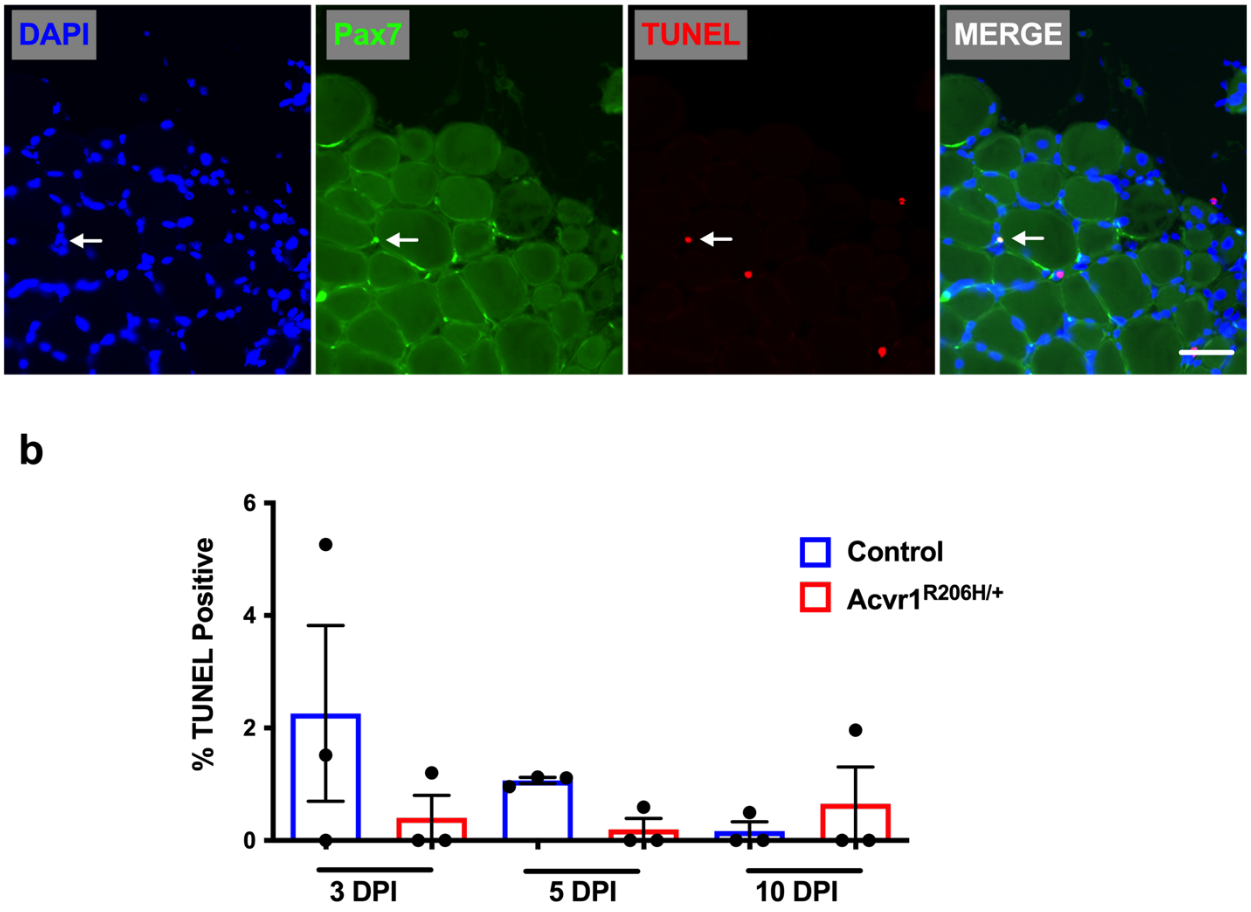

Supplementary Figure 4. Differentiation defect in *Acvr1*$^{R206H/+}$ MuSCs is not due to cell death.

a) Representative images skeletal muscle tissue sections stained for terminal deoxynucleotidyl transferase dUTP nick end labeling (TUNEL, red). MuSCs were visualized by Pax7 detection (green) and nuclei by DAPI (blue). Scale bar=50µm. **b)** Quantification of percentages of TUNEL positive MuSCs in each group. All data are expressed as mean ± SEM; n≥ 3 mice per genotype were examined.

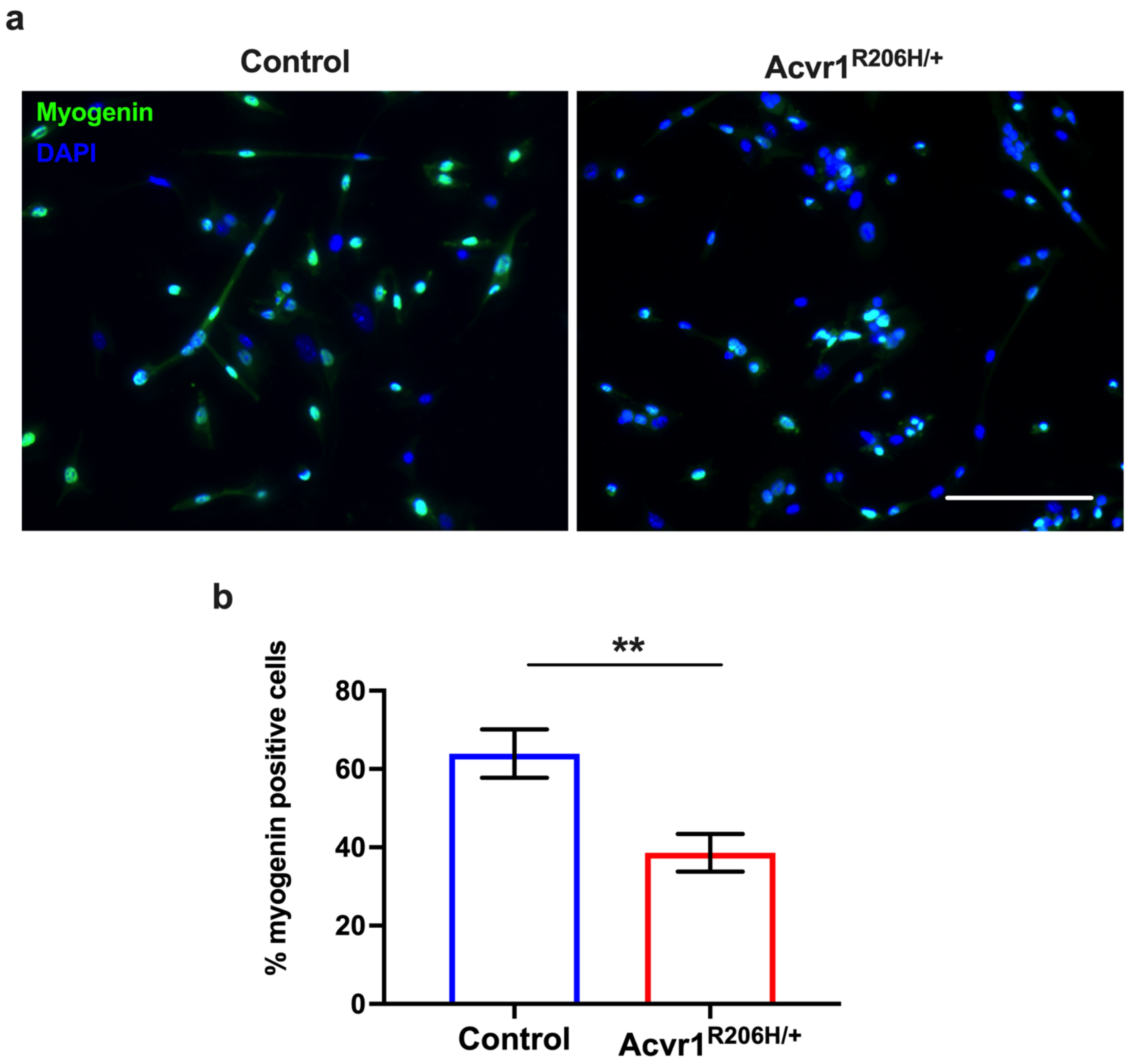

Supplementary Figure 5. Reduced myogenin in *Acvr1$^{R206H/+}$* MuSC*s*

a) Representative images of myogenin (green) staining and DAPI (nuclei, blue) of control and *Acvr1$^{R206H/+}$* MuSCs. **b)** Quantification of % myogenin-positive cells. All data are expressed as mean ± SEM*;* Scale bar=100μm. n=3 mice for each group; N=120-385 cells counted per mouse. Statistical significance was determined by Mann-Whitney test, **p <0.001.

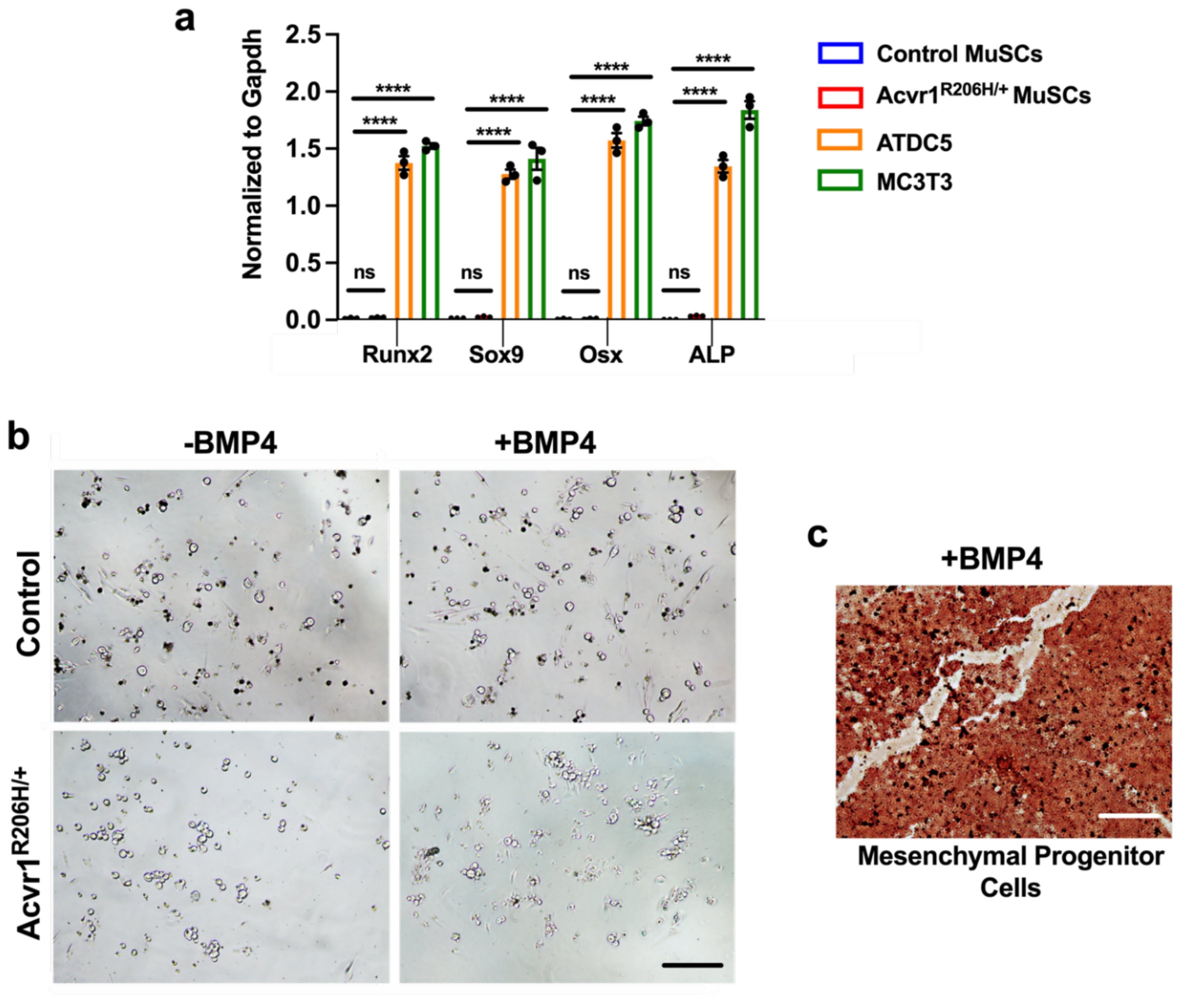

Supplementary Figure 6. Control and *Acvr1*$^{R206H/+}$ MuSCs do not differentiate to HO precursor cells.

a) MuSCs from control and *Acvr1*$^{R206H/+}$ skeletal muscle (uninjured) were isolated by FACS and then examined by qRT-PCR for expression of chondrogenic and osteogenic markers; n=3 mice per genotype per condition. ATDC5 (orange) and MC3T3 (green) cell lines were utilized as positive controls for expression of chondrogenic [Runx2, Sox9] and osteogenic markers [Osterix (Osx), alkaline phosphatase (ALP)]. Graphs represent the mean ± SEM. ****p<0.0001; ns=not significant. Statistical significance was determined using an unpaired t-test. **b**) Representative images of FACS-isolated control and *Acvr1*$^{R206H/+}$ MuSCs cultured for 14 days in osteogenic media with and without BMP4 ligand. Under all conditions, regardless of genotype, MuSCs attached to tissue culture plate but remained unresponsive to osteogenic culture. **c)** In contrast, as a positive control, wildtype murine mesenchymal progenitor cells readily differentiated to mineralizing osteoblasts (detected by Alizarin red staining) following osteogenic culture with BMP4 for 14 days (same culture conditions as in b). Scale bar = 100μM.

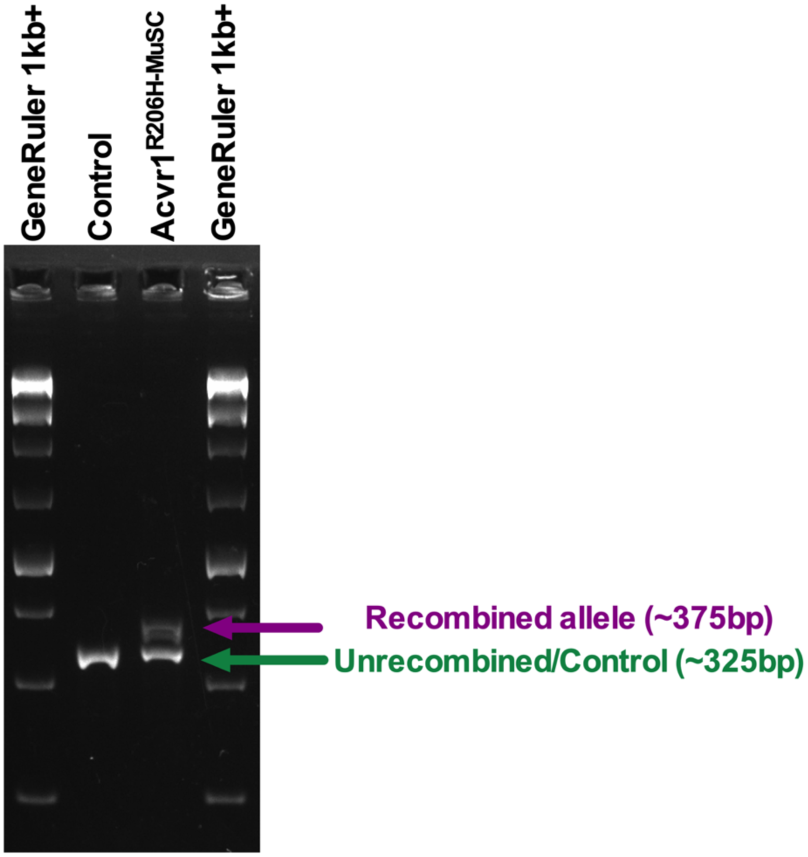

Supplementary Figure 7. Representative image shows the recombination of the *Acvr1$^{R206H/+}$* mouse compared to control (Cre-). Agarose Electrophoresis of PCR products, using skeletal muscle DNA.

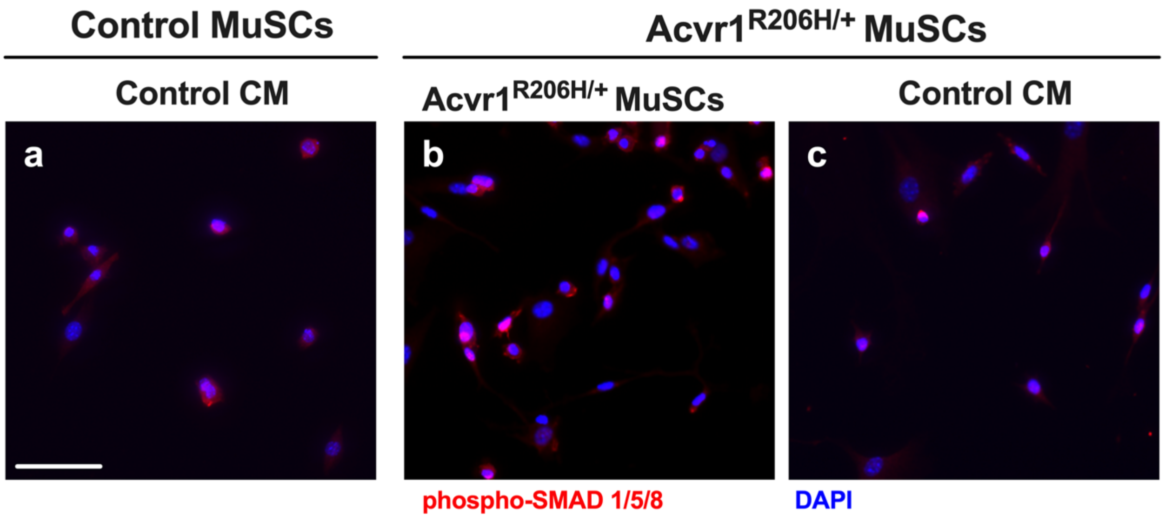

Supplementary Figure 8. Decreased levels of pSmad1/5/8 in *Acvr1$^{R206H/+}$* MuSCs cultured with control conditioned media (CM).

a) Low levels of pSmad1/5/8 in control MuSCs cultured with conditioned media (CM) from control FAPs, **b)** Elevated pSmad1/5/8 protein is detected in *Acvr1$^{R206H/+}$* MuSCs cultured with CM from *Acvr1$^{R206H/+}$* FAPs, **c)** Reduced pSmad1/5/8 levels in *Acvr1$^{R206H/+}$* MuSCs cultured with CM from control FAPs. pSmad1/5/8 is shown in red, and nuclei (DAPI) is shown in blue. Scale bar= 50μm.

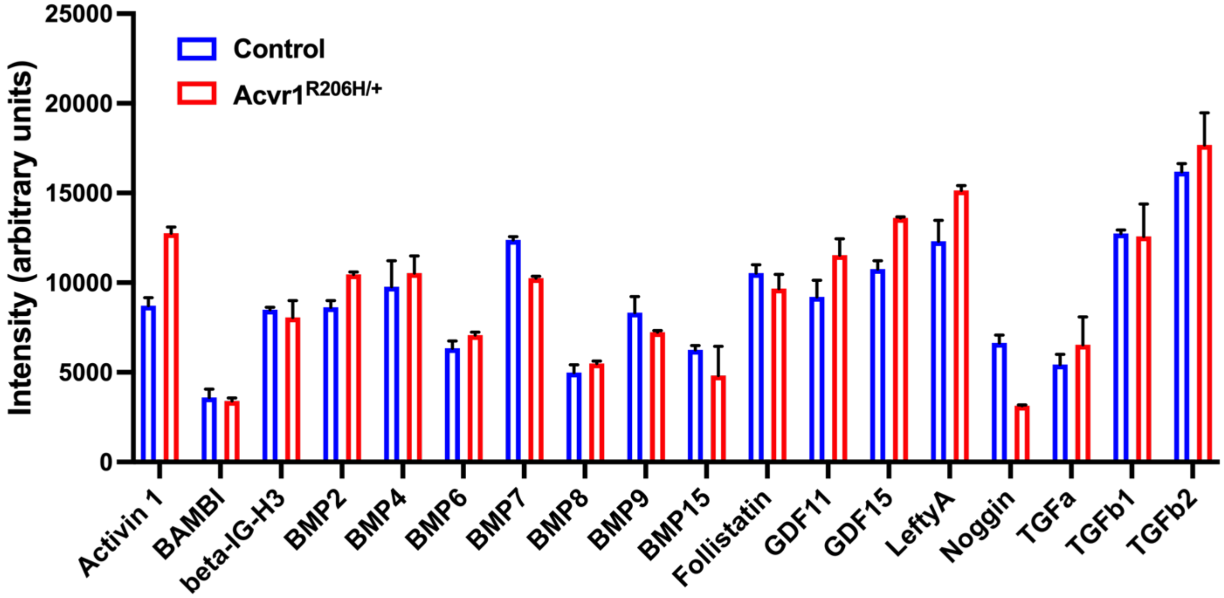

Supplementary Figure 9. Quantification of TGFβ/BMP signaling pathway component proteins in conditioned media (CM) from control and *Acvr1$^{R206H/+}$* FAPs.

CM was collected from FAPs grown in low serum media and total protein levels were equalized before adding to a commercially available protein array (Ray Biotech cat# AAH-TGFB-2). Resulting densitometry is displayed as mean ± SEM.

Supplementary Tables

Supplementary Table 1: MuSC MyoD/Pax7 Expression levels. qRT-PCR data are shown as mean ± SEM.

	$Pax7^{+/}MyoD^{-}$ (%)	$Pax7^{+}/MyoD^{+}$ (%)	$Pax7^{-}/MyoD^{+}$ (%)
Uninjured Control	92.57±0.61	4.57±0.76	3.30±0.30
Uninjured $Acvr1^{R206H/+}$	96.20±0.84	3.51±0.61	0.69±0.70
5 DPI Control	44.18±3.66	32.33±8.45	23.41±2.24
5 DPI $Acvr1^{R206H/+}$	68.30±6.01	32.05±6.06	0.00±0.00

Supplementary Table 2. Antibodies used in FACS.

Antigen	Host	Clone	Conjugate	Source	Dilution
CD45	Rat	30-F11	Biotin	BD Biosciences	1/500
CD11b	Rat	M1/70	Biotin	BD Biosciences	1/200
CD31	Rat	390	Biotin	eBioscience	1/200
Sca1/Ly6A/E	Rat	E13-161.7/D7	Biotin/BV421/BV711	BD Biosciences	1/200
Live/Dead	N/A	N/A	7-aminoactinomycin D (7-AAD)	Sigma Aldrich/Thermo Fisher	1/250
Streptavidin	N/A	N/A	PE-Cy7	Biolegend	1/25
Alpha 7-integrin	Rat	R2F2	AF 647	Ablab	1/25
CD34	Rat	Ram34	BV421	BD Biosciences	1/12.5

Supplementary Table 3. Fluorophores and Aria filter sets.

Fluor	Laser	Filter
7-AAD	Blue	710/50
Alexa Fluor 647	Red	660/20
BV421	Violet	450/50
PE-Cy7	Green	780/60
Sca1/Ly6A/E	Violet	780/40; 450/50
