# Supplementary material for: Dynamics of skeletal muscle-resident stem cells during myogenesis in fibrodysplasia ossificans progressiva
Source: NPJ Regen Med. 2022 Jan 14;7:5. doi: 10.1038/s41536-021-00201-8 (PMC8760285; doi:10.1038/s41536-021-00201-8)
Supplement: Supplementary file 2 — Reporting Summary [file 41536_2021_201_MOESM2_ESM.pdf]

nature research

Corresponding author(s): Eileen M. Shore and Foteini Mourkioti

Last updated by author(s): November 23, 2021

Reporting Summary

Nature Research wishes to improve the reproducibility of the work that we publish. This form provides structure for consistency and transparency in reporting. For further information on Nature Research policies, see our Editorial Policies and the Editorial Policy Checklist.

Statistics

For all statistical analyses, confirm that the following items are present in the figure legend, table legend, main text, or Methods section.

n/a	Confirmed	
☐	☒	The exact sample size (n) for each experimental group/condition, given as a discrete number and unit of measurement
☐	☒	A statement on whether measurements were taken from distinct samples or whether the same sample was measured repeatedly
☐	☒	The statistical test(s) used AND whether they are one- or two-sided<br>*Only common tests should be described solely by name; describe more complex techniques in the Methods section.*
☐	☒	A description of all covariates tested
☐	☒	A description of any assumptions or corrections, such as tests of normality and adjustment for multiple comparisons
☐	☒	A full description of the statistical parameters including central tendency (e.g. means) or other basic estimates (e.g. regression coefficient) AND variation (e.g. standard deviation) or associated estimates of uncertainty (e.g. confidence intervals)
☐	☒	For null hypothesis testing, the test statistic (e.g. F, t, r) with confidence intervals, effect sizes, degrees of freedom and P value noted<br>*Give P values as exact values whenever suitable.*
☒	☐	For Bayesian analysis, information on the choice of priors and Markov chain Monte Carlo settings
☒	☐	For hierarchical and complex designs, identification of the appropriate level for tests and full reporting of outcomes
☒	☐	Estimates of effect sizes (e.g. Cohen's d, Pearson's r), indicating how they were calculated

Our web collection on statistics for biologists contains articles on many of the points above.

Software and code

Policy information about availability of computer code

Data collection	Fluorescent and H&E microscope images: Eclipse TE2000-U inverted fluorescent microscope (Nikon)<br>FACS: FACSDIVA 8+ (BD Biosciences)<br>RT-qPCR: Quantstudio6 Real Time PCR instrument (Applied Biosystems)<br>Micro-computed topography: VivaCT40 imager (Scanco Medical AG, Brüttisellen, Switzerland)
Data analysis	Fluorescent and H&E microscope images: Eclipse TE2000-U inverted fluorescent microscope (Nikon), Photoshop CS6 (Adobe)<br>H&E: Image J<br>BrdU quantification: Image J<br>Micro-computed topography: Scanco µCT V6.1 (Scanco Medical AG, Brüttisellen, Switzerland)<br>FACS: FlowJo 10.1 software (BD Biosciences)<br>TUNEL & RT-qPCR analysis: Prism 7+ (Graphpad)<br>Statistics: Excel 2016+ (Microsoft), Prism 7+ (Graphpad)

For manuscripts utilizing custom algorithms or software that are central to the research but not yet described in published literature, software must be made available to editors and reviewers. We strongly encourage code deposition in a community repository (e.g. GitHub). See the Nature Research guidelines for submitting code & software for further information.

Data

Policy information about availability of data

All manuscripts must include a data availability statement. This statement should provide the following information, where applicable:

- Accession codes, unique identifiers, or web links for publicly available datasets
- A list of figures that have associated raw data
- A description of any restrictions on data availability

No public datasets were utilized

Field-specific reporting

Please select the one below that is the best fit for your research. If you are not sure, read the appropriate sections before making your selection.

☒ Life sciences ☐ Behavioural & social sciences ☐ Ecological, evolutionary & environmental sciences

For a reference copy of the document with all sections, see nature.com/documents/nr-reporting-summary-flat.pdf

Life sciences study design

All studies must disclose on these points even when the disclosure is negative.

Sample size	No sample size calculation was performed. Sample sizes were chosen based on the type of experiment, availability of animals, and standard practice when using individual animals as biological replicates (general range n=3-5).
Data exclusions	No data were excluded from analysis.
Replication	We analyzed biological (not technical) replicates to ensure reproducibility. Experiments were reproducible across different days and investigators involved.
Randomization	Samples were randomly assigned to different conditions, but no attempt was made to control for any other covariates. Different conditions were never batched into different days.
Blinding	Investigators were blinded during all image analysis. Clear cutoffs and automated analyses were used to minimize the effects of investigator bias.

Reporting for specific materials, systems and methods

We require information from authors about some types of materials, experimental systems and methods used in many studies. Here, indicate whether each material, system or method listed is relevant to your study. If you are not sure if a list item applies to your research, read the appropriate section before selecting a response.

Materials & experimental systems

n/a	Involved in the study	
☐	☒	Antibodies
☐	☒	Eukaryotic cell lines
☒	☐	Palaeontology and archaeology
☐	☒	Animals and other organisms
☒	☐	Human research participants
☒	☐	Clinical data
☒	☐	Dual use research of concern

Methods

n/a	Involved in the study	
☒	☐	ChIP-seq
☐	☒	Flow cytometry
☒	☐	MRI-based neuroimaging

Antibodies

Antibodies used

anti-CD31 BD Biosciences 553370 FACS Mouse monoclonal (MEC13.3) 2μg/ml
anti-CD11b BD Biosciences 749864 FACS Mouse monoclonal (M1/70) 2μg/ml
anti-CD45 BD Biosciences 566168 FACS Mouse monoclonal (30-F11) 5μg/ml
anti-Sca1 BD Biosciences 741708 FACS Mouse monoclonal (D7) 5μg/ml
7-aminoactinomycin D (7-AAD) Sigma Aldrich/Thermo Fisher (A1310) 4μg/ml
PE-Cy7 Streptavidin 557598 BD Biosciences 25μg/ml
anti-alpha 7 integrin 750 Ablab (R2F2) 25μg/ml
Alexa fluor 647 anti-CD34 560230 BD Biosciences (RAM34) 12.5μg/ml
Pax7 Polyclonal antibody PA1-117 Thermo Fisher Scientific 1/50
MyoD Developmental Studies Hybridoma Bank (D7F2) 1/250

Goat anti-rabbit Alexafluor 647 MABF413 Mouse monoclonal Sigma (17A2) 1/250
Alexafluor 488 streptavidin 405235 Biolegend 1/250
5-Bromo-2'-deoxy-uridine Labeling and Detection Kit I 11296736001 Roche
Click-iT Plus TUNEL Assay for In Situ Apoptosis Detection, Alexa Fluor 647 kit C10619 Thermo Fisher Scientific
MyHC Developmental Studies Hybridoma Bank, clone BF-G6; 1/10
Alexa Fluor 488-conjugated goat anti-mouse IgG ab150113 Life Technologies 1/500
Phospho-Smad (pSmad) 1/5/8 (Ser463/465) 9516 Cell Signaling 1/200
Goat anti-rabbit Alexa Fluor 555 secondary antibody A27039 Thermo Fisher Scientific 1/500

Validation

anti-CD31 was validated on mouse splenocytes by the manufacturer and has been characterized in its use for FACS-isolating mouse muscle stem cells in Liu 2013 Nature Protocols.
anti-CD11b was validated on mouse splenocytes by the manufacturer and has been characterized in its use for FACS-isolating mouse muscle stem cells in Liu 2013 Nature Protocols.
anti-CD45 was validated on mouse splenocytes by the manufacturer and has been characterized in its use for FACS-isolating mouse muscle stem cells in Liu 2013 Nature Protocols.
anti-Sca1 was validated on mouse bone marrow myeloid cells by the manufacturer and has been characterized in its use for FACS-isolating mouse muscle stem cells in Liu 2013 Nature Protocols.
7-aminoactinomycin D (7-AAD) was validated by the manufacturer and has been characterized in its use for FACS-isolating of mouse muscle stem cells in Tichy 2018 Skeletal Muscle.
PE-Cy7 Streptavidin was validated by the manufacturer and has been characterized in its use for FACS-isolating of mouse muscle stem cells in Tichy 2018 Skeletal Muscle.
anti-alpha 7 integrin was validated by the manufacturer and has been characterized in its use for FACS-isolating of mouse muscle stem cells in Tichy 2018 Skeletal Muscle.
Alexafluor 647 anti-CD34 was validated by the manufacturer and has been characterized in its use for FACS-isolating of mouse muscle stem cells in Tichy 2018 Skeletal Muscle.
Pax7 Polyclonal antibody was validated by the manufacturer and has been characterized in its use for fluorescent staining of mouse muscle stem cells in Tichy 2018 Skeletal Muscle.
MyoD antibody was validated by the manufacturer and has been characterized in its use for fluorescent staining of mouse muscle stem cells in Tichy 2018 Skeletal Muscle.
Goat anti-rabbit Alexafluor 647 antibody was validated by the manufacturer and has been characterized in its use for fluorescent staining of mouse muscle stem cells in Tichy 2018 Skeletal Muscle.
Alexafluor 488 streptavidin was validated by the manufacturer and has been characterized in its use for fluorescent staining of mouse muscle stem cells in Tichy 2018 Skeletal Muscle.
5-Bromo-2'-deoxy-uridine Labeling and Detection Kit I was validated by the manufacturer and has been characterized in its use for fluorescent staining to examine proliferation of fibroblasts in Vasseur 2002 Oncogene.
Click-iT Plus TUNEL Assay for In Situ Apoptosis Detection kit was validated by the manufacturer in kidney tissue and has been characterized for its use in mouse skeletal muscle tissue in Tichy 2018 Skeletal Muscle.
MyHC antibody was validated by the manufacturer and has been characterized in its use for FACS-isolating of mouse muscle stem cells in Tichy 2018 Skeletal Muscle.
Alexafluor 488-conjugated goat anti-mouse IgG was validated by the manufacturer and has been characterized in its use for fluorescent staining of mouse muscle stem cells in Tichy 2018 Skeletal Muscle.
Alexafluor 555-conjugated goat anti-mouse IgG was validated by the manufacturer and has been characterized in its use for fluorescent staining of various cell types in mice in various publications.
Phospho-Smad (pSmad) 1/5/8 (Ser463/465) was validated by the manufacturer and has been characterized has been characterized in its use for fluorescent staining of various cell types in mice in various publications.

Eukaryotic cell lines

Policy information about cell lines

Cell line source(s)

ATDC5 and MC3T3 cells were obtained from Sigma.

Authentication

ATDC5 and MC3T3 cells were authenticated by Sigma.

Mycoplasma contamination

All cell lines tested negative for Mycoplasma.

Commonly misidentified lines
(See ICLAC register)

We did not use any cell lines listed as misidentified in the ICLAC register.

Animals and other organisms

Policy information about studies involving animals; ARRIVE guidelines recommended for reporting animal research

Laboratory animals

A conditional-on knock-in mouse model Acvr1[R206H]FlEx was developed to encode the common R206H mutant allele in FOP following recombination by Cre by Regeneron Pharmaceuticals. The mouse model used in these studies has previously been reported in: Chakkalakal et al (2016). JBMR 31(9):1647-1651 and Hatsell et al (2015). Sci Transl Med. 7(303):303ra137. The Acvr1[R206H]FlEx mouse can be obtained through a material transfer agreement with Regeneron Pharmaceuticals, Tarrytown, NY. (See Acknowledgements in Hatsell et al (2015). For doxycycline-inducible global allele expression, Acvr1[R206H]FlEx/+ mice were mated with mice double transgenic for R26-rtTA and tetO-Cre (heterozygous Gt(ROSA)26Sortm1(rtTA*M2)Jae and hemizygous Tg(tetO-Cre)1Jaw; Jackson Laboratories) to generate Acvr1[R206H]FlEx/+;Gt(ROSA)26Sortm1(rtTA*M2)Jae; Tg(tetO-Cre)1Jaw mice (which we refer to as Acvr1R206H/+). To induce recombination and global expression of the mutant allele, 4-week-old mice were provided a doxycycline diet chow (625mg/kg, Envigo RMS Inc., TD 01306) for five consecutive days.

Wild animals

The study did not involve wild animals.

Field-collected samples	The study did not involve samples collected from the field.
Ethics oversight	Animal procedures were approved by the University of Pennsylvania IACUC guidelines.

Note that full information on the approval of the study protocol must also be provided in the manuscript.

Flow Cytometry

Plots

Confirm that:

- [x] The axis labels state the marker and fluorochrome used (e.g. CD4-FITC).
- [x] The axis scales are clearly visible. Include numbers along axes only for bottom left plot of group (a 'group' is an analysis of identical markers).
- [x] All plots are contour plots with outliers or pseudocolor plots.
- [x] A numerical value for number of cells or percentage (with statistics) is provided.

Methodology

Sample preparation	Described in Methods section "MuSC and FAP isolation".
Instrument	Described in Methods section "MuSC and FAP isolation".
Software	Collection: FACSDIVA 8+ (BD Biosciences); Analysis: FlowJo 10.1 software (BD Biosciences)
Cell population abundance	Described in the paper in Table 1.
Gating strategy	Described in paper in Methods section "MuSC and FAP isolation" and Figure 2a.

- [x] Tick this box to confirm that a figure exemplifying the gating strategy is provided in the Supplementary Information.
